# Adaptation Begins at the Wall: Hormonal Control of Cell-Wall Plasticity Under Abiotic Stresses

**DOI:** 10.3390/biom16101489

**Published:** 2026-10-13

**Authors:** Nooral Amin, Jiao Jiao, Guangyuan Ma, Lifei Liu, Naveed Ahmad, Jian Wei

**Affiliations:** 1 Agricultural College, Shihezi University, Shihezi 832003, China; aminagric965@gmail.com (N.A.); j2023j@126.com (J.J.); 2 Key Laboratory of Xinjiang Phytomedicine Resource and Utilization, Ministry of Education, Institute for Safflower Industry Research of Shihezi University, Pharmacy College of Shihezi University, Shihezi 832003, China; mgy011103@163.com (G.M.); 13944401269@163.com (L.L.); 3 Northern Key Laboratory of Saline-Tolerant Soybean Breeding, Ministry of Agriculture and Rural Areas, Faculty of Agronomy, Jilin Agricultural University, Changchun 130118, China

**Keywords:** abiotic stress, cell-wall integrity, phytohormones, FERONIA, pectin, cellulose, lignin, suberin

## Abstract

The plant cell wall functions as both a mechanical framework and a dynamic environmental sensor, with its remodeling tightly coordinated by phytohormone signaling and cell-wall integrity (CWI) surveillance. This review synthesizes how hormonal networks regulate wall processes including cellulose deposition, pectin modification, and lignification translating extracellular perturbations via receptor kinases and second messengers into adaptive growth responses. We argue that abiotic stress resilience does not stem from uniformly reinforced walls, but from the capacity to dynamically tune wall mechanics in a spatiotemporally precise manner. Evaluating emerging tools like spatial multi-omics and precision genome editing, we propose a translational roadmap to engineer context-dependent wall plasticity. Integrating causal genetics with field-level phenotyping will be essential for developing climate-resilient crops that maintain yield stability under fluctuating environments.

## 1. Introduction

### 1.1. The Cell Wall as a Living Boundary Between Plant and Environment

Plants are sessile organisms and cannot escape unfavorable changes in water availability, salinity, temperature, oxygen status, nutrient supply, or soil chemistry. Survival therefore depends on rapid and reversible reprogramming of growth, metabolism, transport, and development. The plant cell wall occupies a unique position in these responses because it surrounds virtually every plant cell and must simultaneously provide mechanical strength, permit controlled expansion, maintain cell–cell adhesion, regulate apoplastic transport, and transmit information from the extracellular environment to the protoplast. Modern cell-wall biology consequently treats the wall as a dynamic and signaling-active extracellular matrix rather than a passive shell [1,2,3]. Primary cell walls are composed mainly of cellulose microfibrils embedded in hydrated networks of hemicelluloses and pectins, together with structural and enzymatic proteins. Secondary walls, especially in vascular and supporting tissues, generally contain greater amounts of cellulose and hemicellulose and frequently undergo extensive lignification. The resulting wall is mechanically unusual: it must withstand large tensile forces generated by turgor while selectively yielding in specific directions to permit growth. Wall mechanics therefore emerge from polymer composition, nanoscale organization, cross-linking, hydration, wall pH, and the activity of wall-modifying proteins rather than from the abundance of any single component [3,4].

Abiotic stress directly perturbs the physical variables to which the wall is coupled. Drought and salinity alter water potential and turgor; heat and cold modify membrane properties, enzyme activity, and wall-associated metabolism; flooding changes oxygen availability and tissue hydraulics; and nutrient imbalance or toxic metals can modify wall composition, ion binding, and root growth. These effects generate changes in wall tension, hydration, pH, Ca^2+^ availability, polymer interactions, and redox state. Stress adaptation therefore requires plants to adjust the mechanical properties of the wall while maintaining the structural continuity needed for cellular integrity [5,6]. The wall is also part of an environmental surveillance system. Cell-wall integrity pathways monitor disturbances in cellulose, pectin, wall stiffness, and the mechanical relationship between turgor pressure and the extracellular matrix. Receptor-like kinases, wall-associated proteins, peptides, mechanosensitive channels, Ca^2+^ signals, and ROS contribute to this surveillance. Wall remodeling is therefore both an output of stress signaling and a potential source of information that feeds back to growth and hormone pathways [2,7,8].

### 1.2. Phytohormones at the Crossroads of Growth and Stress Adaptation

Abiotic stress creates a fundamental allocation problem in which plants must protect cellular integrity and conserve resources without permanently sacrificing growth and reproduction. provide the regulatory flexibility needed to solve this problem. ABA, auxin, ethylene, brassinosteroids (BRs), gibberellins (GAs), cytokinins (CKs), jasmonates (JAs), salicylic acid (SA), and strigolactones (SLs) interact with ROS, Ca^2+^, peptide signaling, transcription factors, and metabolic pathways to regulate stress acclimation. Importantly, hormone functions are context-dependent. ABA is strongly associated with water-deficit responses, for example, but auxin, ethylene, BRs, GAs, and CKs can either promote or restrict growth depending on tissue identity, stress intensity, and interactions with other signals [9,10]. The cell wall is one of the principal downstream targets of these hormonal networks. Auxin can rapidly activate plasma-membrane H^+^-ATPases, acidify the apoplast, and favor expansin-mediated wall relaxation. BR signaling regulates cellulose-related genes and participates in feedback from altered pectin status. ABA promotes root barrier formation and can control secondary-wall and lignin programs through SnRK2-dependent phosphorylation of transcriptional regulators [11,12,13]. Ethylene can stimulate expansin-dependent escaping growth during flooding or contribute to growth restriction and wall reprogramming under other stresses [14]. JA and SA are activated by some forms of cell-wall perturbation and connect structural damage with defense and reinforcement responses [15,16]. Hormone-wall relationships are bidirectional. A hormone can modify wall composition or mechanics but altered wall integrity can feed back into hormone biosynthesis, signaling, and sensitivity. THE1, for instance, links wall stiffness with ABA production, while FERONIA (FER) interacts with ABA, auxin, ethylene, JA, and BR pathways at multiple levels. This feedback architecture helps prevent uncontrolled growth: a growth-promoting hormonal signal can only be executed safely when the wall can accommodate it, and structural perturbation can redirect hormonal programs toward repair or reinforcement [9,17]. Collectively, these interactions define a hormone cell-wall mechanochemical axis in which abiotic stress alters wall chemistry and mechanics, CWI systems interpret these changes, and hormone networks regulate adaptive wall states. Within this framework, resilience reflects the capacity to generate an appropriate wall state in the correct tissue and at the correct time rather than maximal wall reinforcement.

### 1.3. Purpose, and Conceptual Framework of the Review

Research on plant hormones and cell-wall biology has often developed along partly separate lines. Hormone-centered studies commonly focus on receptors, kinases, transcription factors, and physiological outputs, whereas cell-wall studies emphasize cellulose, hemicellulose, pectin, lignin, biosynthetic enzymes, wall-modifying proteins, and mechanical properties. Consequently, the mechanistic links connecting environmental stress perception, phytohormone signaling, cell-wall integrity (CWI) sensing, wall remodeling, and stress-adaptive phenotypes remain fragmented across different research areas.

The primary purpose of this review is to integrate these fragmented research domains into a unified mechanistic framework explaining how phytohormone signaling, CWI surveillance, and cell-wall remodeling interact during plant adaptation to abiotic stress. This review differs from a quantitative meta-analysis because it does not statistically pool effect sizes from comparable datasets. Instead, it provides a mechanistic integrative synthesis of molecular, genetic, biochemical, physiological, and biomechanical evidence. Particular emphasis is placed on identifying experimentally supported causal relationships, distinguishing them from associations that remain largely correlative, and evaluating their relevance to crop stress resilience. The review examines how signaling networks involving abscisic acid, auxin, ethylene, brassinosteroids, gibberellins, cytokinins, jasmonates, salicylic acid, and strigolactones interact with CWI receptors, Ca^2+^, reactive oxygen species (ROS), kinase cascades, transcriptional regulators, and cell-wall-modifying enzymes. These interactions are considered across drought, salinity, temperature extremes, flooding, nutrient imbalance, toxic-metal stress, and combined stresses. Evidence from model plants is integrated with available crop studies to assess the extent to which established molecular mechanisms can be translated into agriculturally relevant systems.

**Figure 1:**
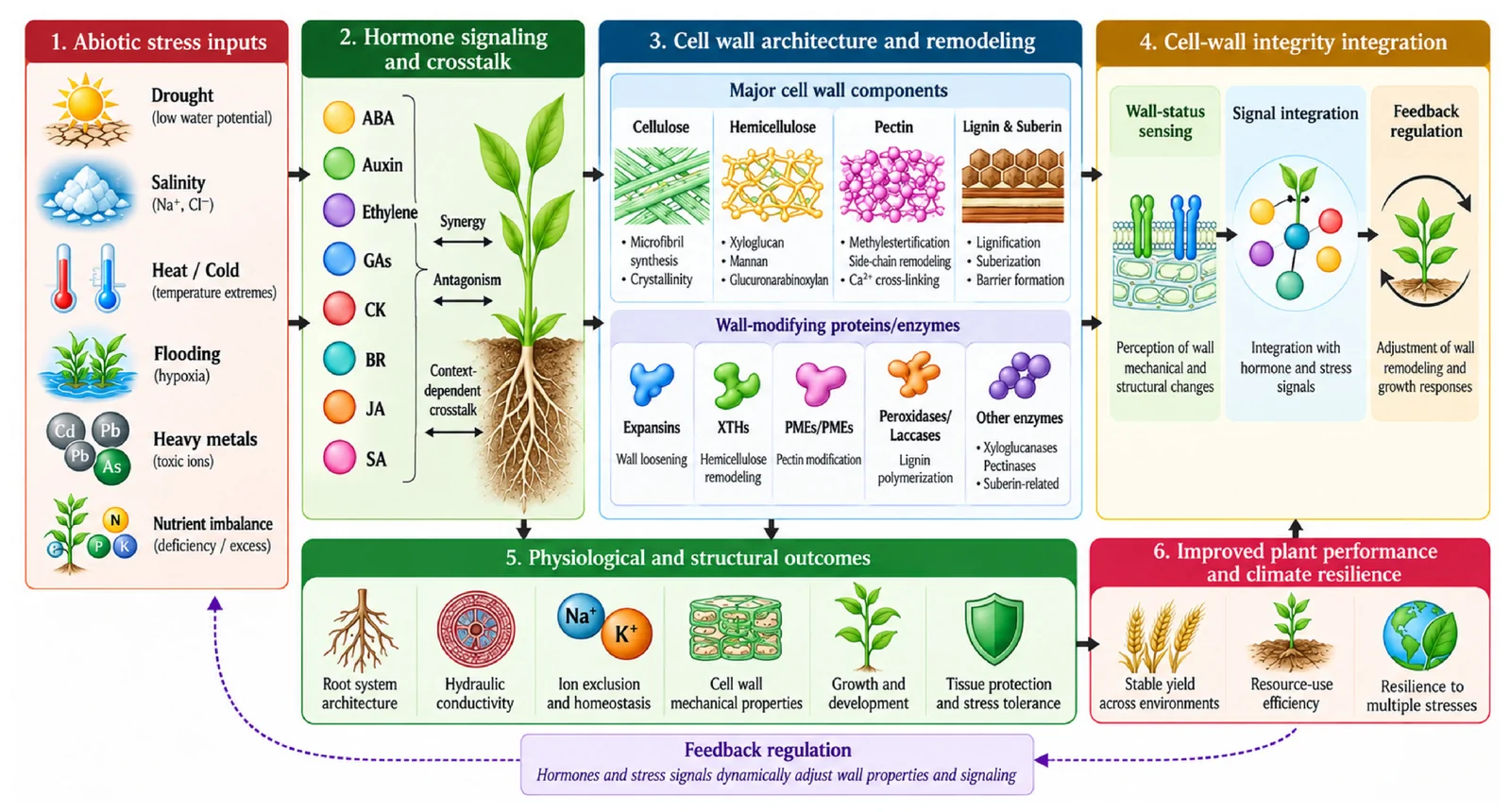
Integrated hormone–cell wall framework for abiotic stress adaptation. Abiotic stresses, including drought, salinity, temperature extremes, flooding, heavy-metal toxicity, and nutrient imbalance, alter hormone signaling and cell-wall properties. Hormonal crosstalk involving ABA, auxin, ethylene, gibberellins (GAs), cytokinins (CKs), brassinosteroids (BRs), jasmonates (JAs), and salicylic acid (SA) regulates cell-wall components and wall-modifying enzymes. Changes in wall structure are integrated through cell-wall integrity sensing, signal integration, and feedback regulation, leading to adjustments in root architecture, hydraulic conductivity, ion homeostasis, wall mechanics, growth, and tissue protection. These coordinated responses contribute to improved plant performance and resilience under multiple abiotic stresses. The feedback loop indicates dynamic adjustment of wall properties and signaling during stress adaptation.

A central proposition of this review is that abiotic-stress tolerance does not depend simply on generating uniformly stronger cell walls. Rather, resilience depends on the capacity to establish appropriate wall states in specific tissues, at particular developmental stages, and under specific environmental conditions through dynamic regulation of wall composition, architecture, mechanics, and barrier properties. Emerging approaches, including spatial multi-omics, mechanobiology, cell-type-specific analyses, epigenetic regulation, and genome engineering, are also considered for their potential to translate mechanistic knowledge into climate-resilient crop improvement. The integrated conceptual framework proposed in this review is summarized in Figure 1. To make this central proposition explicit, the review is organized around a causal framework in which abiotic stress produces environmental or mechanical perturbation, followed by stress and cell-wall integrity (CWI) sensing, hormone and second-messenger signaling, specific cell-wall remodeling, mechanical or transport changes, physiological responses, and ultimately whole-plant performance. The key conceptual advance is that adaptation depends on context-dependent regulation of this sequence rather than on constitutively stronger or weaker walls. Tissue identity, developmental stage, stress intensity, and stress duration determine whether a particular remodeling response is beneficial, neutral, or costly. Importantly, evidence at one level of this sequence should not automatically be interpreted as evidence for the next. Changes in transcript abundance, protein accumulation, or enzyme activity demonstrate molecular or regulatory responses, but do not by themselves establish alterations in wall composition or architecture, changes in wall mechanics or transport properties, or improvements in stress tolerance and whole-plant performance.

Literature Search and Selection Strategy: This study was conducted as a mechanistic integrative narrative review rather than a systematic review or quantitative meta-analysis. Its purpose was to critically synthesize current evidence on the relationships among phytohormone signaling, cell-wall integrity sensing, cell-wall remodeling, and plant responses to abiotic stress. Literature searches were conducted using PubMed/MEDLINE and Google Scholar as the primary discovery sources. These searches were supplemented by literature identified through ScienceDirect, SpringerLink, Wiley Online Library, Taylor & Francis Online, and Frontiers to broaden coverage of relevant studies in plant molecular biology, physiology, cell-wall biology, and abiotic-stress signaling.

Searches used combinations of terms related to abiotic stress, phytohormones, cell-wall metabolism, cell-wall integrity, cellulose, hemicellulose, pectin, lignin, suberin, expansins, xyloglucan endotransglucosylase/hydrolases (XTHs), FERONIA, THESEUS1, wall-associated kinases, RALF peptides, Ca^2+^ signaling, reactive oxygen species, mechanosensing, transcriptional regulation, epigenetic regulation, and crop stress resilience. Stress-specific terms, including drought, salinity, heat, cold, flooding, nutrient imbalance, heavy-metal stress, and combined stress, were also used in different combinations to identify relevant studies. Studies were included when they provided direct or mechanistically informative evidence linking phytohormone signaling, CWI pathways, wall composition, wall-modifying proteins or enzymes, or cell-wall mechanical properties with plant responses to abiotic stress. Priority was given to peer-reviewed primary research providing genetic, molecular, biochemical, physiological, biomechanical, or cell-type-specific evidence. Studies using *Arabidopsis thaliana* and other model species were included when they established mechanisms relevant to conserved signaling pathways, whereas studies in crop species were emphasized whenever available to evaluate translational and agricultural relevance. Studies were excluded when they lacked clear connection to cell-wall regulation or abiotic-stress adaptation, focused exclusively on biotic stress without direct relevance to the mechanisms discussed in this review, provided only descriptive gene-expression data without functional or mechanistic interpretation, or substantially duplicated evidence already represented by more comprehensive or recent studies. Review articles were used primarily to identify major conceptual developments and additional relevant literature, whereas mechanistic conclusions were preferentially supported by original research articles. Because the available studies differ considerably in plant species, developmental stage, tissue type, stress intensity and duration, experimental design, and measurements of cell-wall composition and mechanics, quantitative pooling of effect sizes was not considered appropriate. The evidence was therefore synthesized qualitatively according to the strength of mechanistic support, biological context, relevance to cell-wall regulation, and potential significance for improving crop resilience under abiotic stress.

## 2. Engineering Adaptation at the Cell Wall: Architecture, Remodeling, and Stress Resilience

### 2.1. Major Cell-Wall Components: Cellulose, Hemicellulose, Pectin, and Lignin

The plant cell wall is a heterogeneous extracellular matrix whose mechanical properties arise from interactions among cellulose, hemicelluloses, pectins, lignin, proteins, and water. Cellulose provides the main load-bearing network, and microfibril orientation strongly influences anisotropic growth and tissue mechanics [3,18]. Hemicelluloses vary among species and wall types; xyloglucan is prominent in many eudicot primary walls, whereas xylans are major components of secondary and grass walls. Current models emphasize heterogeneous cellulose–matrix interactions rather than uniformly distributed tethers, so local polymer rearrangements can strongly affect wall extensibility [3,19]. Pectins are especially dynamic because their mechanical effects depend on post-secretory modification. PME-mediated demethylesterification of homogalacturonan can promote Ca^2+^-dependent cross-linking and stiffening, but can also increase susceptibility to pectin-degrading enzymes. Its outcome therefore depends on spatial patterning, apoplastic pH, Ca^2+^ availability, enzyme context, and tissue state [20,21]. Lignin, deposited mainly in secondary walls, increases stiffness, hydrophobicity, and resistance to collapse. Stress-induced lignification can reinforce vascular tissues and diffusion barriers, but excessive deposition restricts expansion and consumes carbon, making its adaptive value strongly dependent on location and timing [22,23,24,25].

These polymers function as an integrated mechanical system, so the relevant stress phenotype is the emergent wall state rather than the abundance of a single component [5,7]. A key consideration for crop translation is the distinction between eudicot Type I and grass Type II primary walls. In many eudicots, xyloglucan and pectin dominate the matrix, whereas grasses such as rice, wheat, maize, barley, and sorghum contain more glucuronoarabinoxylan (GAX) and mixed-linkage (1→3), (1→4)-β-glucan, with ferulate-mediated cross-links contributing substantially to matrix properties [7,26]. In maize, salinity alters arabinoxylan feruloylation and lignin composition, illustrating that cereal wall responses cannot be interpreted through a purely pectin-centered framework [27,28]. Mechanisms established in Arabidopsis should therefore be treated as testable hypotheses rather than direct templates for cereals, where GAX substitution, feruloylation, and mixed-linkage glucan turnover require explicit consideration. To avoid overgeneralization, the mechanisms discussed in the following sections are considered in the context of cell wall type. Some processes, such as cellulose synthesis and organization, secondary-wall lignification, suberization, and CWI-mediated feedback, are broadly shared across angiosperms, although their relative importance can vary among tissues and species. Other mechanisms are more wall-type specific. For example, PME-mediated pectin demethylesterification, Ca^2+^ cross-linking, and xyloglucan remodeling by XTHs are best established in pectin- and xyloglucan-rich Type I walls and may not play the same dominant role in Type II grass walls. In cereals, GAX substitution and feruloylation, mixed-linkage glucan turnover, and xylan–lignin interactions may contribute more strongly to wall mechanics [7,21,26,27,28]. Therefore, throughout this review, findings from Arabidopsis are used as useful mechanistic references rather than as direct models for cereal crops, and the same wall-remodeling process is not assumed to produce an identical mechanical outcome in every species.

### 2.2. The Molecular Foundations of a Living Wall

Cell-wall construction integrates polymer synthesis, trafficking, secretion, and spatial organization. Cellulose is synthesized at the plasma membrane by CESA-containing cellulose synthase complexes (CSCs), whereas most hemicelluloses and pectins are assembled in the Golgi and secreted to the wall [1,29,30]. CSC trajectories are coordinated with cortical microtubules, and their abundance at the cell surface is regulated by secretion, endocytosis, and recycling, emphasizing that cellulose deposition depends on both catalytic activity and membrane trafficking [31]. Abiotic stress can disrupt this machinery by altering cortical microtubules, membrane behavior, and CSC localization; recovery therefore requires restoration or reconfiguration of cellulose deposition [32]. *CESA* transcript abundance alone is consequently insufficient to infer a mechanical wall phenotype. After secretion, matrix polymers also remain dynamic: XTHs restructure xyloglucan, PMEs modify homogalacturonan, and pectin can undergo further enzymatic cleavage. Wall biosynthesis and remodeling therefore occur concurrently [21,29].

Secondary-wall formation coordinates cellulose, xylan, and lignin deposition through NAC/MYB transcriptional networks and extracellular laccase and peroxidase activity. Spatial patterning is as important as polymer quantity because vascular tissues must reinforce selected domains while preserving specialized structures such as pits [33]. Under stress, wall deposition can also be redistributed among tissues and developmental zones, for example by increasing barrier-related polymers in mature roots while maintaining extensibility in elongating regions. Mechanistic studies should therefore integrate gene expression with polymer chemistry, localization, and biomechanical measurements [18].

### 2.3. The Molecular Machinery of Cell-Wall Plasticity

Rapid adaptation also depends on remodeling existing wall architecture. Expansins, XTHs, PMEs, pectin-degrading enzymes, peroxidases, laccases, and β-glucanases modify wall compliance, polymer structure, cross-linking, and lignification [1,21]. Expansins promote stress relaxation and wall extension without hydrolyzing major wall polysaccharides, and their activity is favored by acidic apoplastic conditions, linking auxin-driven proton extrusion with wall mechanics [34]. Their adaptive value, however, depends on where continued expansion is beneficial. XTH proteins remodel xyloglucan, but XET and XEH activities should not be used interchangeably, and XTHs are not universally wall-loosening because different family members can promote restructuring, integration, or strengthening [35,36]. PMEs likewise have context-dependent effects: dimethyl esterification can promote Ca^2+^-mediated associations or create substrates for polygalacturonates and pectate lyases, altering adhesion, porosity, compliance, and signaling [20,21].

Class III peroxidases and laccases contribute to oxidative wall chemistry and lignification, although oxidative wall chemistry can support either reinforcement or loosening depending on context [22,23]. β-Glucanases are also functionally diverse, ranging from enzymes involved in cellulose organization to those acting on callose or mixed-linkage glucans; stress responses should therefore be interpreted according to enzyme family and substrate rather than broad labels such as “cellulase” [37]. Together, these enzymes form a coordinated remodeling network regulated by apoplastic pH, Ca^2+^, ROS, and hormone signaling.

### 2.4. Reshaping Cell Wall Mechanics for Environmental Adaptation

Stress-responsive wall plasticity can be considered in terms of four overlapping processes: loosening, stiffening, lignification, and suberization. Loosening supports continued or redirected growth, whereas stiffening limits excessive expansion and stabilizes tissues. Under drought, for example, root elongation may be maintained while shoot expansion is restricted, requiring different wall states in different organs [5,38]. Importantly, the same biochemical process can have contrasting mechanical outcomes: PME activity can promote Ca^2+^-mediated stiffening or facilitate pectin degradation, while ROS can drive either cross-linking or cleavage [21]. Lignification and suberization provide more persistent forms of structural strengthening and transport regulation. Lignin strengthens vascular and support tissues but excessive deposition can restrict growth and divert carbon, favoring spatially and temporally controlled rather than constitutive reinforcement [24,39]. Suberin forms hydrophobic barriers in the endodermis and, in many crops, the exodermis, thereby regulating radial movement of water and solutes in response to drought, salinity, and nutrient status [40,41]. ABA-responsive MYB factors regulate environmentally induced endodermal suberization in Arabidopsis [42], while a suberized exodermis contributes to drought tolerance in tomato [43]. These examples reinforce the central principle that stress adaptation depends on reallocating wall and barrier properties across tissues rather than simply making walls stronger. The principal remodeling modules and representative hormone/stress connections are summarized in Table 1.

### 2.5. Translating Cell-Wall Remodeling into Plant Function

A recurring problem in stress-related cell-wall research is the tendency to treat molecular, biochemical, mechanical, and physiological observations as interchangeable. These represent distinct levels of evidence and should be distinguished when evaluating causal relationships. In this review, a molecular or regulatory phenotype refers to changes in gene expression, protein abundance or modification, receptor activity, and second-messenger signaling. A biochemical or structural wall phenotype requires direct evidence of changes in enzyme activity, polymer abundance or composition, pectin methylesterification, cross-linking, lignin or suberin deposition, cellulose organization, or related wall architecture. A mechanical or transport phenotype requires direct measurement of properties such as wall stiffness, extensibility, creep, strain, porosity, hydraulic conductance, or ion permeability, whereas physiological and whole-plant phenotypes include cell expansion, stomatal movement, root growth, ion homeostasis, stress recovery, biomass accumulation, and yield [3,18,21,34,53,54]. These levels are causally connected but are not automatically equivalent. For example, altered PME expression does not by itself demonstrate wall stiffening because the mechanical outcome of pectin demethylesterification depends on its spatial pattern, apoplastic pH, Ca^2+^ availability, and the activities of pectin-degrading enzymes [20,21]. Likewise, increased XTH or expansin expression cannot be assumed to produce net wall loosening without evidence of enzyme activity and wall behavior, and greater lignin or suberin abundance does not by itself establish increased wall stiffness, altered transport properties, or improved stress performance [36,39,40,41]. Moreover, measurements of stiffness in living tissues should be interpreted together with hydration and turgor because the measured mechanical response reflects contributions from both wall properties and cellular pressure [53,54]. Therefore, throughout this review, terms such as wall loosening, stiffening, reinforcement, and improved barrier function are considered directly demonstrated only when supported by appropriate biochemical, biomechanical, or transport measurements; otherwise, the proposed downstream mechanical or physiological consequence is explicitly treated as an inference.

## 3. Sensing Cell-Wall Perturbation Under Abiotic Stress

Cell-wall integrity (CWI) signaling is best viewed as a distributed network in which FERONIA, THESEUS1, WAKs, MIK2, RALF peptides, Ca^2+^, ROS, and MAPKs cooperate to translate extracellular wall perturbations into intracellular growth and stress responses. This network architecture allows wall status to be integrated with mechanoperception and hormone signaling rather than assigned to a single universal sensor [2,8].

### 3.1. Cell-Wall Integrity Sensing and Mechanoperception

Cell-wall integrity sensing refers to the capacity of plant cells to detect perturbations that threaten the functional relationship among wall structure, turgor pressure, and growth and to initiate compensatory responses. It overlaps with mechanoperception but is not identical to it. Mechanoperception includes responses to touch, compression, membrane tension, bending, and mechanical impedance, whereas CWI surveillance focuses on whether the extracellular matrix can continue to support growth and cellular integrity [2,55,56]. This distinction matters under abiotic stress because many stresses change both chemistry and mechanics. Salinity alters ionic conditions, pectin interactions, water relations, and wall stiffness; drought changes turgor and tension within load-bearing networks; heat affects wall polymers, membranes, and enzyme activities; and mechanical impedance directly increases forces at the root surface. The cell can therefore infer wall status from multiple signals, including polymer conformation, pectin methylesterification, extracellular peptide availability, receptor organization, plasma-membrane tension, and ion fluxes [6,8]. CWI signaling is best understood as a distributed network rather than a single receptor pathway. Receptor-like kinases including FER, THE1, MIK2, and WAKs, together with RALF peptides, LRX proteins, mechanosensitive channels, Ca^2+^, ROS, and MAPKs, contribute overlapping functions. These pathways can induce wall reinforcement, hormone production, changes in growth, and transcriptional reprogramming. Because wall remodeling changes the stimulus that initiated the response, CWI regulation is inherently feedback based. To distinguish direct receptor evidence from inferred mechanosensing, the evidence discussed below is considered at three levels: (i) direct ligand–receptor or receptor–wall interaction, where biochemical or structural studies demonstrate binding between a defined extracellular molecule and a receptor; (ii) functional CWI evidence, where genetic or physiological experiments show that a receptor is required for a response to wall perturbation; and (iii) inferred mechanical sensing, where receptor activity is associated with wall stiffness, turgor, deformation, or mechanical stress but a direct force-coupled sensing mechanism has not been demonstrated. Direct binding does not by itself prove mechanosensing, and a receptor-dependent stress phenotype does not by itself identify the physical cue being sensed. Mechanosensitive ion channels should be distinguished from these receptor-kinase pathways because membrane-tension-dependent gating can be tested directly, whereas direct force sensing by FER, THE1, MIK2, or WAKs has not been established.

### 3.2. FERONIA, THESEUS1, MIK2, and Wall-Associated Kinases

FER is among the most intensively studied receptors at the wall-plasma membrane interface. It belongs to the CrRLK1L family and functions in growth, reproduction, immunity, hormone responses, and environmental adaptation [57,58]. Under salt stress, FER is required for maintenance of cellular integrity during recovery. Salt-induced wall softening is associated with cell bursting in fer mutants, while Ca^2+^ and borate treatments that favor pectin cross-linking can alleviate aspects of the phenotype. FER-dependent Ca^2+^ transients accompany the response, providing direct evidence that wall perturbation, FER, and Ca^2+^ signaling are functionally connected [59]. FER also binds demethylesterified pectin through its extracellular region and activates ROP6 signaling in pavement-cell morphogenesis [60]. Although this work addresses development rather than abiotic stress directly, it demonstrates a molecular route through which a native wall polymer can influence receptor activity and intracellular signaling. FER should therefore be viewed as part of a multi-component extracellular sensing module rather than as a universal force receptor acting alone. Accordingly, FER–pectin binding provides direct receptor–wall interaction evidence, whereas FER-dependent maintenance of cellular integrity during salt stress provides functional CWI evidence; neither result alone demonstrates that FER directly senses force or stiffness.

THE1 provides complementary evidence for CWI surveillance. It was originally identified because mutations suppressed growth inhibition and ectopic lignification triggered by cellulose-synthesis defects without restoring cellulose itself [61]. THE1 later emerged as a receptor for RALF34 and as a regulator of wall stiffness, turgor-loss point, and ABA biosynthesis [17,62]. These findings place THE1 at an interface between structural wall status and water-deficit physiology. MIK2 is a leucine-rich-repeat receptor kinase with overlapping but distinct functions. Loss of MIK2 modifies responses to cellulose biosynthesis inhibition, including stress-marker expression, jasmonate accumulation, and lignin deposition, and it influences root growth and salt-stress tolerance [63]. The evidence supports MIK2 as a regulator of wall-damage signaling, although a specific direct wall ligand comparable to FER-pectin binding has not been established. In the same way, RALF34–THE1 recognition represents direct ligand–receptor evidence, whereas THE1-dependent effects on wall stiffness, turgor-loss point, ABA biosynthesis, and responses to cellulose deficiency establish functional coupling without proving that THE1 itself is a direct force sensor. MIK2 currently rests mainly on genetic and physiological evidence because a defined direct wall ligand has not yet been established.

WAKs provide a further route for wall-derived signaling. Their extracellular domains associate with pectic polysaccharides, and WAK2 is required for pectin-induced MAPK activity and transcriptional responses [64,65]. WAK signaling therefore illustrates how changes in pectin state can be translated into kinase activity and gene expression, even though many classic WAK studies concern growth and immunity rather than abiotic stress specifically. Arabinogalactan proteins (AGPs) constitute an additional, often underemphasized, layer of the wall-plasma membrane interface that is relevant to this signaling network. These highly glycosylated, GPI-anchored proteoglycans occupy the periplasmic space linking the wall polysaccharide matrix to the membrane and have been implicated in cell expansion, developmental signaling, and ion buffering at the cell surface; under salinity, AGPs accumulate at the plasma membrane and apoplast in a pattern consistent with a role in modulating extracellular ion balance and mechanoperception [28]. Because AGPs, extensins, and other hydroxyproline-rich glycoproteins are physically interposed between the FER, THE1, WAK, and MIK2 ectodomains and the wall polysaccharide network, they are plausible modulators, and in some context’s candidate co-receptors, of the CWI signaling events described above, even though their specific abiotic-stress functions remain considerably less mechanistically resolved than those of RALF peptides or pectin itself. Extensin arabinosylation has similarly been proposed to stabilize the wall and limit uncontrolled loosening under ionic stress. Given the pace of AGP and extensin research in other developmental contexts, direct tests of AGP involvement in FER- and THE1-dependent stress signaling represent a tractable near-term extension of the CWI framework. WAK–pectin association and pectin-dependent WAK2 signaling therefore provide direct evidence for extracellular polymer recognition, but they should not be equated with direct sensing of wall tension or deformation.

### 3.3. RALF Peptides and Receptor-Mediated Wall Signaling

RALF peptides form a family of secreted regulators that connect extracellular conditions with receptor-kinase signaling, apoplastic pH, growth, and stress responses. RALF1 binds FER and inhibits primary-root cell expansion. It also promotes phosphorylation of the plasma-membrane H^+^-ATPase AHA2 at an inhibitory site, contributing to extracellular alkalinization and reduced wall expansion [66]. This mechanism provides a direct biochemical connection between peptide perception and the chemical environment of the wall. RALF perception often involves heteromeric complexes rather than a single receptor. Structural analysis of RALF23 signaling showed that LORELEI-like GPI-anchored proteins can bind RALFs and facilitate assembly of RALF-LLG-FER complexes [67]. LRX proteins, which are strongly associated with the wall matrix, also bind selected RALFs. Under salt stress, LRX3/4/5, RALF22/23, and FER form a functionally connected module that influences plant growth and salt tolerance [68].

This module also intersects with hormone signaling. Perturbation of LRX-RALF-FER function alters ABA-, JA-, and SA-associated responses and changes the expression of numerous salt-responsive genes [69]. RALFs therefore help bridge wall-associated proteins, receptor kinases, and hormone networks. Nevertheless, RALFs should not be described as universal direct mechanosensors. A more defensible interpretation is that they modulate the sensitivity and output of receptor complexes operating at the wall-plasma membrane interface. RALF specificity is important. RALF34 acts as a ligand for THE1, whereas RALF1 and RALF22/23 are linked to different FER-containing modules. Differences in ligand abundance, processing, receptor composition, pH, and wall state could therefore encode distinct extracellular conditions. The 2026 synthesis of cell-wall remodeling and peptide signaling further highlights small secreted peptides as an expanding layer of stress-associated wall regulation, while also emphasizing the need for functional validation of many proposed peptide roles [70]. Thus, defined RALF–receptor interactions can be considered direct ligand–receptor evidence, whereas assigning these receptor complexes a mechanosensory role requires separate mechanical perturbation and biophysical validation.

### 3.4. Ca^2+^, ROS, ROP GTPases, and MAPK Signaling

Ca^2+^ is one of the earliest second messengers activated by mechanical and abiotic stress. Stress-specific Ca^2+^ signatures differ in amplitude, duration, localization, and frequency, allowing common ionic machinery to encode diverse inputs. Mechanosensitive channels, receptor-regulated transport systems, and membrane depolarization can all contribute to these signatures [56]. In the FER salt-stress system, spatially defined Ca^2+^ transients are required for maintenance of wall integrity, establishing a direct link between CWI signaling and Ca^2+^ dynamics [59]. ROS provide a second rapid signaling layer. Controlled production by NADPH oxidases and wall-associated redox enzymes participates in growth and stress responses, whereas excessive accumulation causes oxidative damage. FER functions upstream of ROPGEFs and ROP GTPases in root hairs, with downstream effects on NADPH oxidase-dependent ROS production [71]. Pectin-dependent FER activation of ROP6 provides an additional route from wall chemistry to ROP signaling [60].

Ca^2+^ and ROS signaling can amplify one another. Ca^2+^-dependent protein kinases can activate NADPH oxidases, while ROS can alter ion-channel activity. At the whole-plant scale, interacting Ca^2+^ and ROS waves contribute to systemic signaling under heat, high light, salinity, and wounding [72]. CWI pathways may initiate or modulate some of these responses, but systemic stress signaling also receives inputs from many non-wall sensors, so causal attribution must be conservative. MAPKs provide another route from receptor activation to transcriptional change. Pectin-dependent MAPK activity requires WAK2, demonstrating direct coupling between extracellular pectin status and kinase signaling [64]. Yet there is no single universal MAPK cascade downstream of all CWI receptors. FER, THE1, MIK2, and WAKs share outputs but also use distinct partners. MAPKs are therefore best understood as integration nodes within broader CWI, hormone, and immune networks. This systemic Ca^2+^-ROS-wave evidence is explicitly whole-plant in scale and is therefore distinguished from receptor mechanisms inferred from isolated biochemical assays, seedlings, mutants, or inhibitor-treated material.

### 3.5. From Mechanical Perturbation to Transcriptional Responses

The adaptive significance of CWI sensing lies in its ability to convert rapid changes in cell-wall status into sustained transcriptional, biochemical, and structural responses. Perturbation of cellulose biosynthesis activates THE1-dependent transcriptional programs associated with defense responses, oxidative stress protection, and wall reinforcement [61]. Similarly, WAK2 mediates pectin-dependent gene activation, whereas MIK2 contributes to stress-responsive signaling, jasmonate accumulation, and lignification following cellulose perturbation [63,64]. Downstream second messengers further enhance signaling specificity: Ca^2+^ signatures are decoded by calmodulin-related proteins, calcium-dependent protein kinases, and CBL–CIPK modules that regulate ion transport, metabolic processes, and transcriptional regulators. In parallel, ROS and MAPK cascades modulate transcription factor activity and chromatin-associated responses, thereby linking extracellular wall perturbations with long-term cellular reprogramming. Importantly, CWI signaling does not function as an independent mechanical surveillance pathway but is integrated with hormonal regulatory networks that coordinate growth, defense, and wall adaptation. THE1-associated signaling influences ABA-related responses, whereas LRX–RALF–FER modules interact with multiple hormone pathways to regulate stress responses and developmental transitions [9,17]. Thus, hormone signaling and CWI perception operate through a reciprocal feedback network in which hormonal pathways regulate wall biosynthesis and remodeling, while changes in wall composition and mechanical state are integrated through CWI-associated pathways that activate Ca^2+^-, ROS-, and kinase-dependent signaling to adjust downstream transcriptional and hormonal responses. For many receptor systems, however, the specific extracellular cue and the extent to which mechanical information is sensed directly remain unresolved.

**Figure 2:**
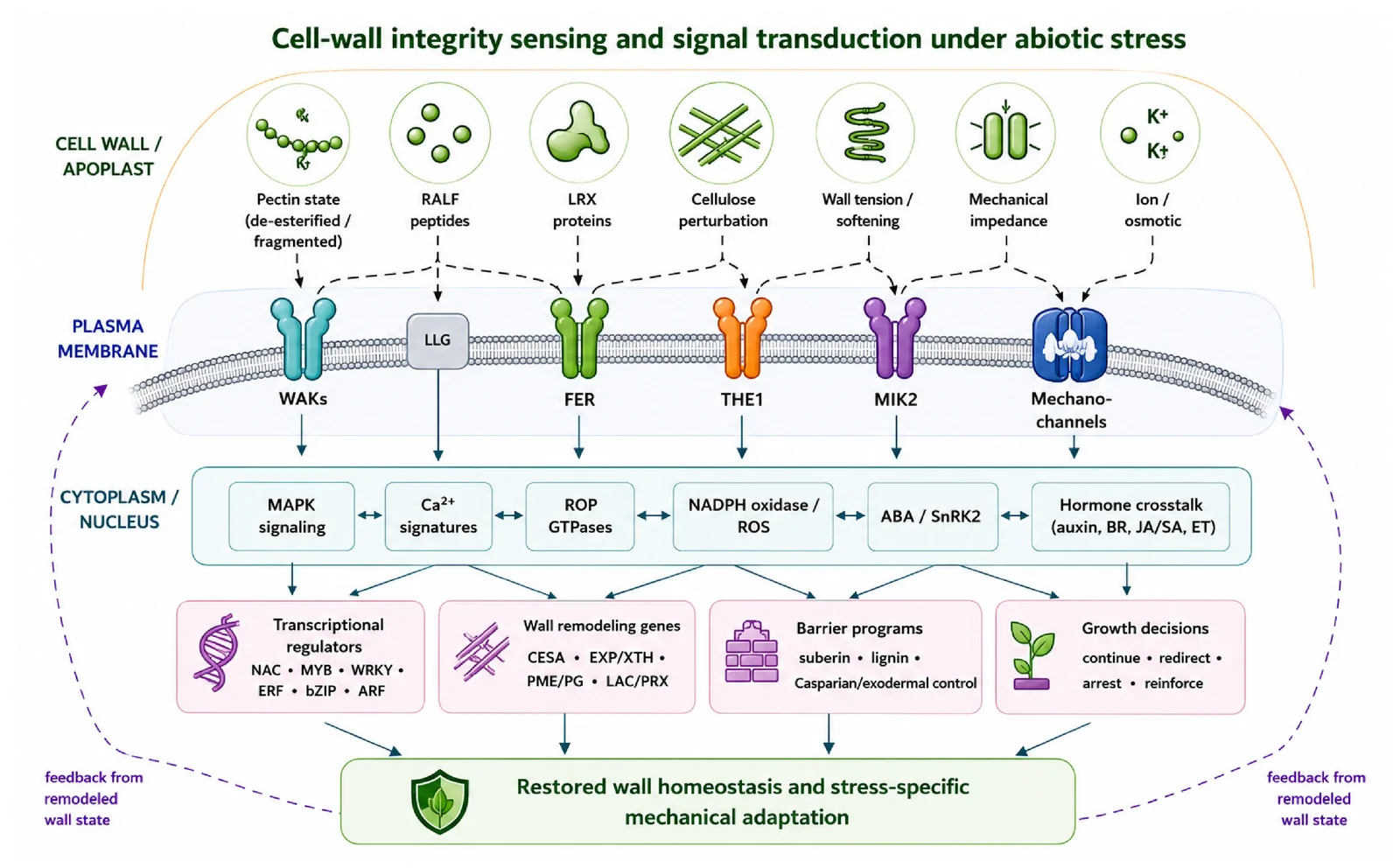
Cell-wall integrity sensing and signal transduction under abiotic stress. Environmental stresses modify extracellular wall properties, including pectin status, RALF/LRX signaling, cellulose organization, wall tension, mechanical impedance, and ionic conditions. These changes are perceived by plasma membrane-localized CWI sensors, including WAKs, FER, THE1, MIK2, and mechanosensitive channels, which activate interconnected MAPK, Ca^2+^, ROP GTPase, ROS, and hormone signaling pathways. Downstream transcriptional and biochemical programs regulate wall remodeling, including cellulose synthesis, expansin/XTH-mediated restructuring, pectin modification, oxidative cross-linking, and barrier formation through lignin, suberin, and Casparian strip pathways. Feedback from remodeled wall states to CWI signaling enables dynamic adjustment of growth, reinforcement, and mechanical adaptation, allowing plants to maintain wall homeostasis and stress resilience.

The specificity of these adaptive decisions is likely determined by the combination of receptor composition, ligand availability, Ca^2+^ signatures, ROS distribution, tissue identity, developmental stage, and pre-existing hormonal status. Despite substantial advances, important limitations remain. Much of the current mechanistic understanding is derived from Arabidopsis seedlings, cellulose-biosynthesis inhibitors, acute salt treatments, or developmental systems, leaving uncertainty regarding how conserved these mechanisms are under realistic agricultural conditions. Crop plants exposed to progressive drought, fluctuating salinity, heat waves, soil compaction, or combined stresses may employ distinct receptor combinations and wall-state transitions. Future translational studies will therefore require integration of live mechanical measurements, quantitative wall chemistry, genetically encoded signaling reporters, cell-type-specific perturbation approaches, and spatial transcriptomics in crop organs. Such approaches will be essential for determining how CWI–hormone networks generate adaptive wall states that balance growth, protection, and productivity under complex environmental conditions. The integrated CWI signaling architecture and its major downstream wall-remodeling outputs are shown in Figure 2.

## 4. Phytohormonal Control of Cell-Wall Remodeling

Phytohormones coordinate cellular responses to environmental perturbations by regulating cell expansion, wall biosynthesis, barrier formation, and tissue differentiation. Their effects are strongly context-dependent because the same hormone can promote expansion in one tissue while favoring reinforcement or growth restriction in another. The most useful organizing principle is therefore not a fixed “wall function” for each hormone, but rather the set of wall processes that a hormone can regulate and the environmental, developmental, and tissue contexts in which those processes become adaptive [9,10]. Accordingly, the hormone-centered sections below are interpreted using the same evidence hierarchy established above. Regulatory changes involving CESA, PME, XTH, expansin, lignin, or suberin-related pathways are distinguished from directly measured changes in wall composition or architecture, and both are distinguished from mechanical or transport phenotypes. Where the literature links a hormone to gene expression, protein activity, or polymer abundance but does not directly measure stiffness, extensibility, permeability, hydraulic conductance, or related physical properties, the mechanical consequence is described as inferred rather than demonstrated. Similarly, a measured change in wall mechanics or transport is not assumed to confer stress tolerance unless it is linked experimentally to physiological or whole-plant performance. This distinction is essential because the same molecular remodeling process can generate different physical and physiological outcomes depending on tissue identity, developmental stage, stress intensity, duration, and the surrounding biochemical and mechanical environment.

### 4.1. Abscisic Acid: Wall Reinforcement, Lignification, Suberization, and Water-Deficit Adaptation

ABA is central to drought and osmotic-stress responses and controls stomatal behavior, growth, transcription, and root hydraulics. Direct evidence also connects ABA signaling to secondary-wall formation. SnRK2.2, SnRK2.3, and SnRK2.6 phosphorylate the NAC regulator NST1, promoting expression of downstream genes associated with cellulose and lignin biosynthesis; impairment of ABA biosynthesis or signaling reduces secondary-wall thickness and lignification in Arabidopsis [13]. This establishes a causal route from canonical ABA signaling to wall reinforcement. ABA is also a major regulator of root suberization. MYB41, MYB53, MYB92, and MYB93 form a partially redundant module required for much of environmentally induced endodermal suberin deposition, and several members respond to ABA. In crop roots, related barrier systems can be equally important. Tomato requires a functional suberized exodermis for normal drought adaptation, illustrating that crop-specific root anatomy must be considered when translating Arabidopsis mechanisms [43].

ABA additionally influences primary-wall mechanics in specialized cells. Guard-cell pectin remodeling affects the ability of stomata to translate turgor changes into aperture changes, and ABA-regulated PME activity contributes to this mechanical system. This connection is supported by *PME34* and *PME53* studies linking pectin remodeling with stomatal mechanics, ABA responsiveness, and heat acclimation [73,74]. More broadly, ABA-driven changes in lignification, suberization, pectin state, and growth restriction should be viewed as coordinated regulation of hydraulic and mechanical properties rather than independent outputs. The wall also feeds back to ABA. THE1 links wall stiffness with ABA production and turgor regulation, showing that ABA can function both upstream and downstream of CWI signaling [17]. Thus, water-deficit adaptation depends on a reciprocal ABA-wall system that modifies structure while monitoring whether the resulting wall state remains mechanically appropriate.

### 4.2. Auxin: Apoplastic Acidification, Wall Loosening, and Adaptive Growth

Auxin has one of the clearest direct relationships with wall mechanics. Cell-surface TMK signaling rapidly activates plasma-membrane H^+^-ATPases, increasing proton extrusion and lowering apoplastic pH. Acidification favors wall-loosening processes, including alpha-expansin activity, and supports turgor-driven cell expansion [11,34]. This provides a mechanistic chain from hormone perception to changes in wall physicochemistry within minutes. Auxin also regulates pectin chemistry. It influences expression and local activity of pectin-remodeling enzymes, pectin deposition, and the mechanical properties of developmental domains. Consequently, auxin does not simply soften the wall, it can reorganize pectin in spatially precise ways that alter adhesion, stiffness, and organ patterning [75]. Under abiotic stress, auxin redistribution helps reshape root architecture. Drought, salinity, nutrient deficiency, and mechanical impedance alter auxin biosynthesis, transport, and signaling, which in turn regulate primary-root elongation, lateral-root initiation, and root-hair growth. These developmental outputs require localized changes in wall extensibility. Yet global enhancement of auxin-driven loosening would be maladaptive under severe stress because continued expansion increases water and carbon demand. Stress tolerance therefore depends on localized and temporally controlled auxin-wall responses. Auxin signaling is also constrained by CWI feedback. FER and related pathways interact with auxin transport and signaling, while pectin-FER-ROP circuits can modify cell polarity. This relationship helps ensure that auxin-induced growth proceeds only when wall integrity can be maintained.

### 4.3. Ethylene: Wall Remodeling During Stress-Induced Growth Reprogramming

Ethylene is particularly important when stress requires rapid reprogramming of growth. Flooding provides the clearest case because restricted gas diffusion causes ethylene to accumulate rapidly in submerged tissues. Depending on species, ethylene can promote quiescence or escape growth. In the semiaquatic species *Rumex palustris*, ethylene stimulates proton extrusion, apoplastic acidification, *RpEXPA1* transcription, expansin abundance, and expansin activity during submergence-induced petiole elongation [14]. This is direct evidence that ethylene can mobilize wall-loosening machinery to support adaptive growth. Ethylene can also connect environmental stress to specific wall-remodeling genes. Under cadmium stress, EIN3 regulates *XTH33* and contributes to root-growth inhibition, demonstrating a causal ethylene-XTH link in stress-induced growth reprogramming. In other words, ethylene promotes lignification, ROS-related wall responses, or growth cessation. Its wall output therefore depends on whether a tissue is executing escape growth, restricting expansion, or reinforcing structure. Flood-adapted rice illustrates the importance of ethylene crosstalk. Ethylene-responsive factors activate GA-dependent elongation in deepwater rice, linking flood perception with a broader growth program that requires coordinated wall deposition and extensibility [76]. Ethylene should therefore be understood as a regulator that selects among alternative wall-growth programs rather than as a simple promoter of loosening.

### 4.4. Brassinosteroids: Cellulose Synthesis and Wall-Growth Coordination

BRs promote cell expansion and vascular development and are tightly connected to cell-wall biosynthesis. BR-deficient and BR-insensitive mutants exhibit altered cellulose content, and BES1 binds upstream regions of several CESA genes, providing a direct route from BR signaling to cellulose synthesis [77]. More recent synthesis across species confirms that BRs influence cellulose, hemicellulose, pectin, and lignin, emphasizing their role in coordinating wall architecture with growth and environmental state [78]. BR signaling also participates in feedback from the wall. Interference with pectin methylesterification can activate BR signaling, and BR pathways contribute to compensatory responses that restore wall homeostasis [12]. This demonstrates that BRs are not merely upstream growth signals; they are embedded in a feedback circuit that senses whether the wall can support growth. Interactions between BR and FER further reinforce this interpretation. FER can affect BR receptor abundance and downstream signaling, while BR status can influence wall-related growth responses. Under stress, this feedback may help reconcile two competing requirements: maintaining enough wall extensibility for growth while preserving structural integrity.

### 4.5. Jasmonate and Salicylic Acid: Stress-Induced Wall Reinforcement and CWI Responses

JA and SA are classically associated with defence signalling but increasing evidence places both hormones within the broader CWI surveillance network. Genetic or pharmacological disruption of cellulose synthesis can trigger extensive hormonal and transcriptional reprogramming, indicating that plants actively perceive perturbations in wall structure rather than responding only to pathogen-derived signals. In Arabidopsis, reduced cellulose synthesis caused by CESA defects induces ectopic lignification and defence-associated signalling, providing early evidence that impaired wall construction activates compensatory reinforcement mechanisms [79]. Similarly, cellulose biosynthesis inhibition by isoxaben induces marked accumulation of JA and SA together with ROS production, lignification and defence-gene expression; importantly, these responses are strongly influenced by carbohydrate availability, turgor status and osmotic support [15]. More recent work has begun to resolve the molecular architecture underlying these responses. Cellulose biosynthesis inhibition activates a SCOOP18-MIK2 peptide receptor module that couples CWI perturbation to pattern-triggered immunity and compensatory lignification [16], reinforcing the concept that wall damage is translated into active immune signalling rather than functioning simply as a structural defect. Consistent with this framework, current models of plant cell-wall signalling place JA, SA, ROS and receptor-like kinase pathways among the major outputs of cellulose-deficiency sensing [80,81].

JA can also act downstream of stress perception to directly modify wall construction. In Arabidopsis, JA activates the MYC2–MYB46 transcriptional module, inducing secondary-cell-wall biosynthetic genes, cellulose synthesis and lignin accumulation, thereby providing a mechanistic connection between jasmonate signaling and structural wall reinforcement [82]. SA likewise interacts with cell-wall-associated defense programmes, although its effects are strongly dependent on tissue and stress context. SA signaling can promote lignin-associated phenylpropanoid responses during abiotic stress, while in developmental contexts it may redirect resources away from secondary-wall differentiation toward immunity. Thus, JA and SA should not be regarded merely as downstream defense hormones; rather, they form part of an integrated CWI–stress signaling network that converts changes in wall status into context-dependent programmes of growth restriction, wall reinforcement and immune activation.

### 4.6. Gibberellins and Cytokinins: Regulation of Wall Extensibility and Growth

GAs promotes growth largely by inducing degradation of DELLA repressors, thereby changing transcriptional networks that control cell expansion, metabolism, and wall deposition. Transient GA3 treatment can drive rapid anisotropic hypocotyl expansion, demonstrating that GA-dependent growth requires coordinated changes in wall mechanics and deposition rather than simple elastic stretching [83]. During flooding, ethylene-GA crosstalk drives dramatic internode elongation in deepwater rice, illustrating how GA-dependent wall growth can become an adaptive survival strategy. CKs provide a complementary control system. In Arabidopsis roots, cytokinin promotes cessation of cell expansion in the shootward part of the elongation zone, accompanied by increased wall stiffness and dependence on auxin transport [84]. Hormone-regulated expansin studies show that altering *EXPA1* can change wall biomechanics, pectin methylesterification, and expression of other wall-remodeling genes, cautioning against the assumption that higher expansin abundance always makes a wall softer [85]. Together, GAs and CK illustrate that hormones define where growth continues and where it stops. Under stress, their balance with ABA, auxin, and ethylene determines whether a tissue maintains expansion, transitions to differentiation, or adopts a more conservative mechanical state.

### 4.7. Strigolactones and Emerging Hormonal Regulators of Wall Adaptation

SLs regulate shoot branching, root architecture, nutrient acquisition, and stress responses. Direct evidence linking SLs to wall remodeling is more limited than for ABA, auxin, ethylene, or BRs, and this difference in evidence strength should be explicit. In cotton fibers, SLs promote fiber elongation and secondary cell-wall thickening, supporting a direct role for SL signaling in wall development [86]. This demonstrates that SLs can influence both expansion and secondary-wall deposition in a specialized crop cell. During drought, salinity, and nutrient limitation, SLs alter root architecture, stomatal behavior, redox state, and interactions with ABA, auxin, CK, ethylene, and GAs. These developmental outputs are likely to involve cell-wall remodeling, but a changed root phenotype is not equivalent to a defined biochemical wall mechanism. Future studies should therefore test whether SL-dependent stress adaptation modifies CESA dynamics, pectin methylesterification, expansin/XTH activity, lignification, suberization, or CWI signaling in specific cell types. Other signaling metabolites and peptide hormones are also emerging as regulators of wall adaptation. Their inclusion is most useful when direct links to wall chemistry or mechanics are demonstrated rather than inferred from general stress tolerance. The expanding evidence for RALF-family peptides is particularly important because it connects extracellular ligands with FER/THE1 signaling and hormone crosstalk [70]. Representative hormone wall mechanisms, biological contexts, and evidence anchors are briefly summarized in Table 2.

## 5. Hormone Crosstalk Governing Cell-Wall Plasticity

Hormone pathways rarely act in isolation under abiotic stress. Changes in one hormone can modify the biosynthesis, transport, sensitivity, signaling, or downstream transcriptional responses of another, and these interactions are superimposed on CWI, peptide, Ca^2+^, ROS, and metabolic signals. Cell-wall plasticity is therefore an emergent output of an interconnected regulatory network rather than the sum of independent hormone effects. This network view is essential because the same hormone can produce contrasting wall outcomes depending on tissue identity, developmental stage, stress intensity, and stress phase [9,10]. To make these interactions mechanistically clear, crosstalk is evaluated at seven separable levels: (i) hormone concentration and biosynthesis, (ii) transport and spatial distribution, (iii) hormone sensitivity, (iv) receptor and downstream signaling competence, (v) transcriptional or post-translational regulation, (vi) biochemical or structural cell-wall remodeling, and (vii) mechanical, transport, or physiological output. Crosstalk can occur at any of these levels; therefore, a change in hormone abundance is not assumed to imply altered signaling, a defined wall phenotype, or improved stress performance. Mechanistic interpretation requires evidence linking the relevant regulatory step to the corresponding wall and physiological consequences.

### 5.1. ABA-Auxin Coordination

ABA and auxin often exert contrasting effects on growth, but their interaction is better described as coordinated spatial regulation than simple antagonism. During drought or salinity, ABA commonly promotes stomatal closure, root barrier formation, and growth restraint, whereas changes in auxin transport, distribution, and sensitivity determine which root zones continue elongating and where lateral roots or root hairs develop. The adaptive phenotype therefore depends on the spatial partitioning of ABA and auxin outputs rather than on whole-organ hormone concentrations alone [10,75]. Water deficit can suppress shoot growth while maintaining or redirecting root growth, requiring localized wall yielding in elongating tissues together with increased suberization or lignification in more mature regions. ABA-responsive MYB factors contribute to barrier formation, whereas auxin-dependent proton extrusion and expansin activity support localized expansion. CWI signaling can further modify this balance through THE1-dependent regulation of ABA and FER-associated interactions with auxin signaling. Mechanistically, these regulatory levels should be distinguished. Water deficit can enhance ABA accumulation and signaling while altering auxin transport and distribution, but local auxin output depends on transport, tissue sensitivity, and signaling competence rather than bulk indole-3-acetic acid concentration alone [10]. At the signaling level, TMK-dependent auxin signaling provides a well-defined example of auxin-to-wall coupling by activating plasma-membrane H^+^-ATPases and promoting apoplastic acidification [11], whereas ABA-responsive MYB factors regulate environmentally induced root suberization [42]. These mechanisms illustrate how the same root can combine barrier reinforcement in mature tissues with auxin-associated wall yielding in elongating zones. However, changes in hormone abundance, transport, receptor signaling, or transcription should not be interpreted as direct evidence of altered wall stiffness or extensibility unless the corresponding mechanical properties are measured [75]. Overall, ABA–auxin crosstalk therefore represents a spatially and temporally coordinated mechanism that balances protective wall reinforcement with localized growth, allowing roots to adjust development without compromising cell-wall integrity under stress.

### 5.2. ABA-Ethylene Interactions

ABA and ethylene frequently interact to coordinate transitions between water conservation, growth restriction, and stress-induced developmental reprogramming. Under drought, ABA is a central regulator of stomatal closure and hydraulic conservation and promotes root barrier formation through enhanced suberization, whereas ethylene can modify root architecture, restrict elongation under severe stress, or contribute to acclimatory developmental responses [90]. Their interaction is therefore strongly dependent on tissue identity, stress intensity, and developmental context. Under flooding, this relationship changes substantially. Restricted gas diffusion promotes rapid ethylene accumulation, and ethylene subsequently interacts with ABA, gibberellins, auxin, and other hormonal pathways to determine whether tissues adopt escape elongation, growth quiescence, aerenchyma formation, or adventitious-root development [89,91]. At the cell-wall level, ethylene can directly initiate growth-associated remodeling. In submerged *Rumex palustris*, ethylene promotes H^+^ extrusion and apoplastic acidification and increases *RpEXPA1* expression and expansin activity, thereby facilitating the wall yielding required for rapid petiole elongation [14]. By contrast, ABA-associated drought responses favor hydraulic conservation and barrier reinforcement, particularly through regulation of root suberization [90]. Thus, ABA-ethylene crosstalk can generate contrasting wall states depending on whether a tissue must conserve water, restrict growth, or undergo rapid adaptive elongation.

Submergence in *R. palustris* provides a particularly useful time-resolved example in which hormone perception, transcriptional regulation, wall remodeling, and growth can be experimentally separated. Ethylene-dependent H^+^ efflux and apoplastic acidification occur rapidly after submergence, *RpEXPA1* transcript abundance increases within approximately 2 h, expansin protein abundance and activity increase after about 4–6 h, and accelerated petiole elongation becomes evident after approximately 4 h [14]. Inhibition of ethylene perception with 1-methylcyclopropene (1-MCP) suppresses the submergence-induced increase in *RpEXPA1*, demonstrating that this transcriptional response requires ethylene perception. Importantly, *RpEXPA1* regulation is ethylene-dependent rather than mediated directly by ABA or GAs, whereas ABA and GAs participate in parallel ethylene-modulated pathways controlling elongation [14]. Following de-submergence, the hormonal context changes again as reoxygenation, oxidative stress, dehydration, and restoration of growth require coordinated regulation of stress signaling, repair, and recovery processes [92]. Thus, hormone concentration, receptor perception, downstream transcription, biochemical wall remodeling, and physiological recovery represent distinct but interacting levels of the response rather than a single linear signaling cascade.

### 5.3. Auxin-Brassinosteroid Cooperation

Auxin and BRs are strongly interconnected growth regulators. Auxin rapidly modifies apoplastic pH and pectin chemistry, whereas BRs influence *CESA* expression, wall biosynthesis, and wall-homeostasis feedback. Their cooperation therefore links acute modulation of wall extensibility with longer-term adjustment of wall construction [34,75]. At the transcriptional level, auxin- and BR-responsive factors share targets and can cooperate in promoting cell expansion. At the cell surface, both pathways influence plasma-membrane processes and cytoskeletal organization that affect cellulose deposition. Because cell expansion requires synthesis of additional wall material, synergistic auxin-BR signaling can coordinate wall relaxation with continued polymer production. This coordination is particularly important during rapid growth, where loosening without sufficient biosynthesis would compromise integrity. Stress changes this relationship by imposing resource and mechanical constraints. Under salinity or drought, growth-promoting auxin-BR outputs may be restricted to selected tissues, while ABA and CWI pathways prevent excessive expansion. FER is a potential interface because it interacts with both auxin and BR signaling and senses wall-associated cues. Thus, auxin-BR cooperation should be understood as part of a mechanically gated growth program rather than a constitutive synergy.

### 5.4. JA-Ethylene and SA-JA/ABA Interactions

JA, ethylene, and SA interact at multiple mechanistic levels during stress and CWI responses. Perturbation of cellulose synthesis can simultaneously activate JA, SA, ROS, defense-associated transcription, and lignification [15,79]. Quantitatively, 12 h of isoxaben treatment increased JA concentrations by approximately 10^3^–10^4^-fold in Arabidopsis seedlings supplied with glucose or sucrose, whereas osmotic support strongly reduced or abolished this response [15]. This finding illustrates that hormone accumulation alone does not determine the downstream response; wall integrity, turgor, and metabolic context also influence signaling output. At the transcriptional level, JA activates the MYC2–MYB46 module to promote secondary-cell-wall biosynthetic programs [82], whereas ethylene acts through EIN3 to regulate *XTH33* during cadmium-induced root-growth inhibition [88]. These pathways provide distinct mechanistic entry points into wall remodeling, although direct evidence that JA–ethylene crosstalk itself produces a defined mechanical wall phenotype remains limited. Similarly, SA interacts with JA- and ABA-associated redox and phenylpropanoid pathways, but many proposed wall-related effects under heavy-metal stress remain supported primarily by associative rather than direct mechanical evidence [93]. These relationships illustrate a recurring principle: hormone ratios and receptor context are often more informative than absolute hormone abundance. For example, 12 h of isoxaben treatment caused a very large increase in JA, around 10^3^–10^4^-fold, in Arabidopsis seedlings supplied with glucose or sucrose. Importantly, the large JA increase described here was measured in Arabidopsis seedlings subjected to the cellulose-biosynthesis inhibitor isoxaben under defined carbohydrate and osmotic conditions. It is therefore evidence from a seedling/inhibitor system and should not be generalized quantitatively to untreated mature plants.

However, this response was strongly reduced or even abolished by osmotic support, showing that the effect of JA depends on the surrounding turgor, metabolic, and CWI conditions [15]. Receptor context is also important. Auxin can activate TMK-dependent signaling at the cell surface, leading to H^+^-ATPase activation, apoplastic acidification, and wall-associated growth responses [11], but the final outcome still depends on the signaling machinery present in a particular tissue. In the same way, FER connects wall status with several hormone pathways, including ABA, auxin, ethylene, JA, and BR, and its output depends on ligand identity and receptor-complex composition [9]. The LRX–RALF–FER module provides another example of how a specific extracellular signaling complex can influence several hormone-related responses during salt stress [69]. Together, these examples show that hormone concentration alone does not reliably predict the resulting wall response; it should be considered together with receptor and signaling context, CWI and turgor status, tissue identity, and directly measured wall changes [9,11,15,69]. Thus, mechanistic interpretation should distinguish relative hormone abundance, hormone sensitivity and signaling competence, downstream transcriptional responses, CWI status, tissue context, and directly measured wall properties, rather than assuming that hormone concentration or a particular hormone ratio alone predicts the resulting wall phenotype.

### 5.5. FERONIA as an Interface Between Hormone Crosstalk and Wall Integrity

FER is particularly important because it sits at the intersection of extracellular wall status and multiple hormone pathways. Recent synthesis documents bidirectional interactions between FER and ABA, auxin, ethylene, JA, and BR signaling. Hormones can regulate FER abundance, kinase activity, RALF expression, or receptor-complex composition, while FER can modify hormone biosynthesis, transport, perception, and downstream transcription [9]. This architecture provides a plausible mechanism for coordinating growth with mechanical integrity. Auxin or BR may promote expansion, but FER-dependent information about pectin state or wall tension can redirect downstream signaling if the wall is compromised. Under salinity, FER helps maintain wall integrity through Ca^2+^ signaling; through its hormone interactions, the same receptor system can influence longer-term growth and stress programs [9,59]. FER should therefore not be treated as another hormone receptor in a linear pathway. It is better conceptualized as an extracellular-state integrator whose outputs depend on ligand identity, wall composition, RALF/LRX complexes, tissue context, and hormone status. Crop homologs of FER and associated signaling components are promising targets, but direct functional equivalence should be established before translational manipulation because receptor-family expansion and tissue specialization can differ among species. Future studies should move beyond single-hormone manipulation toward network-level regulation. Hormone combinations, receptor sensitivity, spatial hormone distributions, and feedback from wall status should be considered when designing stress-resilient crops. Integration of hormone biosensors, spatial omics, and mechanical measurements will be necessary to establish causal relationships between signaling networks and wall phenotypes.

### 5.6. From Single-Hormone Responses to Multi-Hormone Regulatory Networks

The accumulated evidence argues against designing wall-based stress tolerance around a single hormone. A more realistic model is a multi-hormone control system in which ABA regulates water conservation and barriers; auxin defines local expansion; ethylene selects growth reprogramming strategies; BRs coordinate wall production with growth; JA and SA connect structural perturbation with protective responses; and GAs, CK, and SLs adjust developmental priorities [10,94,95]. Hormonal stress responses are increasingly understood as interacting regulatory networks rather than independent linear pathways. CWI receptors and peptide signals constrain these outputs according to the mechanical state of the wall [2,57]. CrRLK1L receptors such as FERONIA and THESEUS1 participate in cell-wall-integrity surveillance and integrate physical wall status with peptide, hormonal, Ca^2+^, ROS, and growth-related signaling. This network is dynamic across time. Early stress responses may emphasize rapid ion fluxes, apoplastic pH, and wall relaxation; intermediate responses may alter transcription, pectin chemistry, and growth; and prolonged stress may increase lignification, suberization, secondary-wall deposition, or developmental reprogramming [90,95,96]. Auxin-driven acidification and expansin action provide a well-supported example of rapid wall loosening, whereas stress-associated lignification, suberization, and secondary-wall reinforcement represent slower structural responses. Conceptually, this trajectory can be organized into four overlapping phases, including early signaling, intermediate remodeling, prolonged structural adaptation, and recovery. These phases depend on tissue type, stress type, intensity, and duration rather than representing rigid sequential steps. Early signaling, occurring within seconds to minutes, includes changes in hormone perception or distribution, Ca^2+^ and ROS transients, ion fluxes, and apoplastic pH. Intermediate remodeling, developing over minutes to hours, includes receptor-dependent transcriptional and post-translational regulation together with changes in expansin, XTH, and PME activity and in pectin or cellulose organization. Prolonged adaptation, over hours to days, can involve lignification, suberization, secondary-wall deposition, and developmental reprogramming, whereas recovery requires rebalancing hormone signaling, restoring growth and transport, repairing wall-associated damage, and either reversing or retaining selected wall states [14,90,92,95,96]. The sequence is not necessarily linear: fluctuating stress may repeatedly return tissues to early or intermediate phases, whereas under sequential stress a second challenge may occur while hormonal, transcriptional, or structural states established by the first stress are still present. If such retained states persist after recovery and modify a later response, they may contribute to stress memory [97,98,99]. Thus, the outcome of hormone crosstalk depends not only on which hormones are involved, but also on when signaling occurs relative to the current wall state and the plant’s recent stress history.

Hormone crosstalk therefore needs to be studied as a trajectory rather than at a single sampling time. For crop improvement, the network view implies that constitutive overproduction or activation of a stress-hormone pathway may produce undesirable pleiotropy [94,100]. More promising strategies include tissue-specific promoters, stress-inducible regulatory alleles, editing of cis-regulatory elements, or manipulation of receptor interfaces that alter sensitivity only under defined conditions [100,101,102]. Such approaches aim to preserve growth under favorable conditions while enabling rapid stress responses when required. Tissue- or condition-dependent expression can reduce problems associated with constitutive expression, promoter editing can fine-tune gene activity, and engineered ABA receptor agonist systems demonstrate that hormone signaling can be activated conditionally without constitutive growth penalties.

## 6. Stress-Specific Hormone-Cell-Wall Remodeling

### 6.1. Drought and Osmotic Stress

Drought reduces water potential and turgor, forcing plants to reconcile water conservation with continued root function. The cell wall is central to this response because turgor-driven growth is mechanically dependent on wall extensibility. Drought therefore produces spatially contrasting wall states: shoot expansion is often restricted, mature root tissues may increase lignification or suberization, and selected root elongation zones can maintain wall loosening to explore deeper or wetter soil layers [5]. ABA dominates many drought responses and controls stomata, secondary-wall programs, and root barrier formation. ABA-responsive suberin regulators decrease uncontrolled radial water loss and modify hydraulic conductivity, while ABA-NST1 signaling can promote secondary-wall and lignin pathways [13,42]. Crop evidence from tomato shows that exodermal suberin contributes directly to water-deficit tolerance, emphasizing that root barrier anatomy can be as important as endodermal signaling in Arabidopsis [43]. Wall-type context is particularly important when interpreting drought responses. Processes such as cellulose reorganization, lignification, suberization, and adjustment of root apoplastic barriers occur in both eudicots and grasses, but the matrix polymers that control wall extensibility differ. Evidence linking drought responses to PME-dependent pectin demethylesterification or xyloglucan-focused XTH activity is therefore most directly applicable to Type I walls; in Type II cereal walls, analogous mechanical adjustment may rely more strongly on GAX substitution/feruloylation, mixed-linkage glucan turnover, and grass-specific xylan architecture. Thus, pectin- or xyloglucan-based mechanisms identified in Arabidopsis should be tested directly in rice, wheat, maize, barley, or sorghum before being generalized to cereal drought tolerance [7,26,38].

At the same time, auxin, expansins, XTHs, and pectin-remodeling enzymes help preserve localized growth. Recent drought-focused wall synthesis highlights coordinated changes in pectin methyl esterification, expansin and XTH activity, lignification, suberization, guard-cell mechanics, and CWI signaling rather than a single universal wall response [38]. This complexity explains why drought-tolerant genotypes may show either increased or decreased wall stiffness depending on tissue and developmental stage. CWI signaling may also contribute to drought acclimation by monitoring the changing relationship between turgor and wall resistance. THE1 modifies wall stiffness and ABA production, and FER-related signaling can influence hormone pathways and ion fluxes. A priority for future work is to measure wall mechanics and hormone dynamics simultaneously during progressive soil drying, because rapid osmotic treatments do not fully reproduce field drought. Secondary-wall xylan integrity has recently emerged as a further, hemicellulose-centered determinant of drought outcome that complements the lignification- and suberization-focused view above. In Arabidopsis and hybrid aspen, genetically or enzymatically reducing the integrity of secondary-wall glucuronoxylan improved survival, increased stomatal density, and delayed growth inhibition under moderate drought relative to wild-type plants, although the direction and magnitude of the benefit depended on genotype and stress severity, with reduced or reversed advantages under severe water deficit [103]. This finding is notable because it identifies xylan structure itself, rather than lignin content or pectin methylesterification, as a lever connecting secondary-wall integrity signaling to drought physiology, and because it shows that a moderately compromised rather than reinforced wall can sometimes improve short-term drought performance. Because grass walls differ markedly from Arabidopsis secondary walls in xylan substitution and cross-linking, whether this response can be translated to cereals should be tested directly rather than inferred from eudicot models.

### 6.2. Salinity and Ionic Stress

Salinity responses also need to be considered in relation to cell wall type. Pectin demethylesterification can affect wall charge and Ca^2+^ binding in both eudicots and grasses, but pectin makes up a smaller proportion of Type II grass primary walls. As a result, its role in salt-induced wall stiffening and ion buffering may vary more across tissues and species than it does in Arabidopsis. In cereals, salinity can also influence GAX substitution and feruloylation, xylan cross-linking, and lignification. For example, maize shows salt-responsive changes in arabinoxylan feruloylation and lignin composition [27,28]. Therefore, the pectin- and FER-centered mechanisms discussed below should be viewed as well-supported mechanistic examples rather than as responses that occur to the same extent in all cereal cell walls. Salinity combines osmotic stress with ion toxicity and therefore imposes both mechanical and chemical demands on the wall. Early salt exposure reduces water uptake and changes turgor, while prolonged exposure causes Na^+^ and Cl^−^ accumulation, ROS production, and metabolic disruption. Cell walls contribute by controlling mechanical growth, binding ions through charged groups, and regulating apoplastic barriers [28]. Pectin is particularly important because its carboxyl groups interact with Ca^2+^ and other cations. Changes in pectin methylesterification alter charge density, Ca^2+^ cross-linking, wall stiffness, and ion-binding capacity. Salt can also perturb cellulose synthesis and microtubule organization, requiring recovery of CSC behavior for sustained growth [32]. These effects occur alongside XTH, expansin, and lignin changes that differ among tissues and species. FER provides one of the strongest mechanistic links between salinity and CWI. Salt-induced wall softening compromises cell integrity in fer mutants, and FER-dependent Ca^2+^ signaling supports recovery [59]. LRX-RALF-FER modules also regulate salt tolerance and multiple hormone pathways [68,69]. Thus, salinity is a model stress in which wall mechanics, peptide signaling, Ca^2+^, and hormone crosstalk are experimentally connected. Suberin provides a longer-term ion-exclusion strategy. Root suberin lamellae can reduce passive Na^+^ entry and alter radial transport; salt stress changes suberin thickness, composition, and biosynthetic gene expression [41]. The adaptive optimum, however, is not maximal barrier formation because water and nutrient uptake must continue. Salt tolerance therefore requires coordinated control of wall mechanics and selective permeability. The FER result is specifically genetic evidence from fer mutant material under salt treatment; it demonstrates pathway dependence for cellular integrity and recovery, while whole-plant salt tolerance in mature crops requires separate validation.

### 6.3. Heat Stress

Heat stress disrupts membrane stability, protein homeostasis, photosynthesis and respiration, cellular metabolism, growth, and tissue water relations, while simultaneously provoking substantial remodeling of the plant cell wall. Rather than functioning as a static mechanical barrier, the wall undergoes temporally coordinated changes that contribute both to stress perception and to maintenance of cellular integrity. A recent synthesis proposes a biphasic working model in which relatively rapid wall loosening and pectin remodeling facilitate cellular adjustment and stress signaling, whereas prolonged or recovery-phase responses promote restoration or reinforcement of cellulose, hemicellulose, and lignin networks [104]. Such dynamic remodeling is likely important for balancing the need for continued growth with prevention of excessive wall weakening under damaging temperatures. Pectin metabolism appears particularly important in this process. Pectin methylesterases (PMEs) alter the degree of homogalacturonan methylesterification, thereby modifying wall charge, porosity, mechanical properties, and the capacity of pectins to interact with Ca^2+^. In soybean seedlings, heat shock stimulates PME-associated pectin demethylesterification and mobilization of apoplastic Ca^2+^, and restoration of Ca^2+^–pectate interactions is associated with acquired thermotolerance, providing direct evidence that pectin remodeling and Ca^2+^ homeostasis is functionally connected during heat acclimation [105]. In Arabidopsis, PME34 further contributes to heat tolerance by regulating guard-cell wall properties and promoting effective stomatal movement, linking cell-wall remodeling with transpiration-mediated cooling [73]. Similarly, guard-cell-specific PME53 participates in ABA-dependent stomatal regulation and heat responses [74]. These observations are consistent with the broader finding that the methylesterification status of guard-cell pectin determines wall mechanical flexibility and the dynamic range of stomatal opening and closure [106]. The soybean evidence cited here was obtained in seedlings, so it supports a mechanistic link between heat-responsive pectin remodeling, apoplastic Ca^2+^ dynamics, and acquired thermotolerance at the seedling stage. It is distinguished from evidence in mature intact plants and from the Arabidopsis guard-cell studies, which address a specialized cell type and genetic regulation of stomatal mechanics.

Heat-responsive wall remodeling is also closely integrated with cytoskeletal, hormonal, reactive oxygen species (ROS), and Ca^2+^ signaling networks. Elevated temperature can reorganize cortical microtubule arrays through PIF4-dependent regulatory pathways during thermomorphogenic growth, thereby connecting temperature perception with cytoskeletal organization and cell expansion. Phytohormones further shape these responses: ABA promotes water conservation through stomatal regulation and interacts with guard-cell wall remodeling, whereas brassinosteroids, auxin, and ethylene participate in growth adjustment, antioxidant defense, and broader thermotolerance programs [73,74,107]. ROS and Ca^2+^ signals generated during heat exposure can additionally intersect with cell-wall-integrity (CWI) signaling. Importantly, recent evidence has strengthened the mechanistic connection between extracellular wall status and FERONIA (FER) signaling, pectin–RALF interactions can generate extracellular molecular condensates that recruit FER–LLG1 receptor complexes and coordinate stress-responsive cell-surface signaling [108]. Nevertheless, the complete causal sequence linking primary temperature perception to individual hormone-regulated wall-modifying enzymes remains less comprehensively resolved than some established growth-regulatory pathways. A useful experimental framework is therefore to distinguish rapid physical and signaling events from later structural acclimation. Measurements during the first minutes to hours of heat exposure should prioritize apoplastic pH, Ca^2+^ dynamics, PME activity, pectin methyl esterification, wall extensibility, ROS production, and CWI signaling, whereas later stages should assess recovery of cellulose synthesis, hemicellulose organization, pectin status, lignification, stomatal function, and hormone-dependent transcriptional regulation. Such temporal resolution is essential for distinguishing reversible, adaptive wall plasticity from progressive and irreversible heat-induced cellular damage.

### 6.4. Cold and Freezing Stress

Cold and freezing alter membrane phase behavior, enzyme kinetics, water relations, ROS, and extracellular ice formation. Cell walls influence freezing tolerance through elasticity, porosity, water distribution, and the mechanical accommodation of dehydration. Cold acclimation can alter pectin composition, lignification, cellulose-related gene expression, and wall-associated proteins, but responses vary strongly among species and tissues. Hormone signaling provides an important regulatory layer. ABA, JA, ethylene, BRs, auxin, CKs, GAs, and SLs all contribute to cold responses through overlapping networks that regulate antioxidant capacity, growth, CBF/DREB transcription, and development. A recent synthesis emphasizes that cold tolerance emerges from hormonal crosstalk rather than a single dominant pathway [109]. Direct genetic and biomechanical evidence strengthens this wall-centered view: cold acclimation increases pectic β-1,4-galactan and is accompanied by decreased wall extensibility and increased rigidity, whereas galactan-deficient Arabidopsis mutants show impaired acquired freezing tolerance. Galactan-deficient Arabidopsis mutants support a causal role for pectic galactan in freezing tolerance, although validation in intact crop plants under field-like conditions remains limited.

From a wall perspective, the relevant question is how these hormone networks alter mechanical compliance during cellular dehydration. Excessive stiffness could increase damage as cells shrink, whereas uncontrolled loosening could compromise tissue integrity. Pectin methyl esterification and Ca^2+^ cross-linking are plausible control points, while lignification and secondary-wall changes can provide longer-term reinforcement. Crop-specific studies are still needed to establish which of these processes causally determine freezing survival. The cold stress literature therefore illustrates a broader gap, stress-responsive transcription of wall genes is abundant, but direct measurements of wall mechanics under cold and freezing are comparatively scarce. Combining cryo-compatible imaging, AFM or related mechanical assays, and cell-type-specific hormone reporters would significantly strengthen mechanistic understanding.

### 6.5. Flooding, Waterlogging, and Hypoxia

Flooding and waterlogging restrict gas diffusion and reduce oxygen availability, resulting in hypoxia, altered energy metabolism, ROS accumulation, and major hormonal changes. Ethylene rapidly accumulates in submerged tissues and regulates adaptive responses such as aerenchyma formation, adventitious rooting, shoot elongation, and senescence [91,110]. These responses are closely associated with cell-wall remodeling. In *Rumex palustris*, ethylene promotes apoplastic acidification and increases expansin expression and activity, thereby facilitating rapid petiole elongation during submergence [14]. Similarly, in deepwater rice, flooding-induced ethylene interacts with gibberellin signaling to stimulate rapid internode elongation as an escape strategy [111]. In contrast, plants adopting a quiescence strategy suppress excessive elongation and conserve carbohydrates, demonstrating that flooding tolerance can involve either wall loosening or growth restraint depending on the adaptive strategy. Root tissues also undergo substantial structural modification during waterlogging. Aerenchyma formation improves internal oxygen transport, whereas barriers to radial oxygen loss can involve increased suberin and lignin deposition in outer root tissues [110]. Ethylene, ABA, ROS, and Ca^2+^ signaling further coordinate root architectural and metabolic responses during flooding and subsequent recovery [91,110]. Future studies combining cell-wall composition, mechanical measurements, and spatial analyses of hormone and ROS signaling across different crop species will be important for determining which wall modifications directly contribute to flooding tolerance.

### 6.6. Heavy-Metal and Nutrient-Associated Stresses

The cell wall is a major determinant of plant responses to heavy metals because negatively charged groups in pectins, hemicelluloses, lignin, and wall-associated proteins can bind or immobilize metal ions before they enter the cytoplasm. Plants can modify pectin abundance and methylesterification, lignification, and other wall components to regulate metal retention and tolerance [93]. Hormonal and signaling pathways also contribute to these responses. In Arabidopsis, cadmium-induced ethylene signaling stabilizes EIN3, which directly regulates *XTH33*, linking ethylene to cell-wall remodeling and inhibition of root elongation [88]. Salicylic acid and nitric oxide have additionally been implicated in regulating wall-associated heavy-metal responses, while microRNAs can target genes involved in wall biosynthesis and modification [93]. Nutrient deficiencies similarly alter root development and cell-wall organization. Boron is particularly important because it forms borate cross-links between rhamnogalacturonan-II molecules; consequently, boron deficiency disrupts wall integrity, meristem activity, and root development [112]. Phosphate deficiency is also closely linked to apoplastic iron accumulation in the root meristem, which modifies cell elongation and meristem maintenance and contributes to primary-root growth inhibition [113]. Together, these findings demonstrate that the cell wall functions both as a structural matrix and as a chemical interface integrating metal sequestration, nutrient availability, hormone signaling, and root-growth responses. From a comparative perspective, however, metal-associated wall remodeling differs from the predominantly hydraulic challenge imposed by drought and the combined osmotic–ionic challenge imposed by salinity. Increased pectin demethylesterification can expose additional negatively charged carboxyl groups that may enhance apoplastic binding of metal cations, while lignification and other wall modifications can further influence metal retention and transport. However, such changes may also alter wall mechanics, restrict root elongation, and affect water and nutrient acquisition. Similarly, enhanced wall cation-binding capacity may reduce the entry of toxic ions such as Cd^2+^ into the symplast, but extensive sequestration or wall modification may impose physiological costs, particularly when nutrient availability is already limiting. Thus, increased wall sequestration should not automatically be interpreted as improved stress tolerance. Mechanistic studies should distinguish between apoplastic retention, symplastic accumulation, and intracellular sequestration while simultaneously measuring wall chemistry, root mechanics, growth, nutrient status, and whole-plant performance. Such an integrated approach is necessary to determine whether a particular wall modification provides genuine tolerance or primarily redistributes the location and physiological cost of metal toxicity [88,93,112,113].

### 6.7. Combined and Sequential Abiotic Stresses

Field environments expose plants to temporally complex stresses that should not be treated as equivalent. Here, five situations are distinguished: (i) simultaneous combined stress, in which two or more stresses overlap in time; (ii) sequential stress, in which one stress precedes another and the order of exposure may change the response; (iii) fluctuating stress, in which stress intensity or duration repeatedly rises and falls; (iv) stress-recovery cycles, in which stress is interrupted by partial or complete relief before a subsequent stress phase; and (v) stress memory, in which an earlier exposure leaves a persistent molecular, physiological, or epigenetic state that alters a later response after the initiating stress has been removed [97,98,99]. These temporal patterns are biologically distinct because they can recruit different hormone, ROS, Ca^2+^, CWI, transcriptional, and metabolic networks and can therefore generate different wall states rather than a single generic multiple-stress phenotype. Direct evidence for cell-wall remodeling under simultaneous combined stress exists but remains limited. In Arabidopsis, combined heat and osmotic stress altered root wall composition and increased suberization specifically in root regions undergoing secondary development and in lateral roots, whereas the primary-development zone responded differently, demonstrating tissue- and developmental-stage-specific wall remodeling [114]. In cotton fibers, co-occurring elevated temperature and drought reduced cellulose accumulation more strongly than either stress alone and redirected carbohydrate metabolism toward callose at the expense of cellulose synthesis, providing a second direct biochemical and structural example [115]. These studies support non-additive wall responses under simultaneous stress, but neither establishes a complete causal chain from hormone/CWI signaling through direct biomechanical measurements to whole-plant performance. Thus, much of the broader mechanistic interpretation of combined-stress wall behavior still relies on transcriptomic, metabolic, or physiological evidence and on mechanisms established under single stresses [97,116,117].

**Figure 3:**
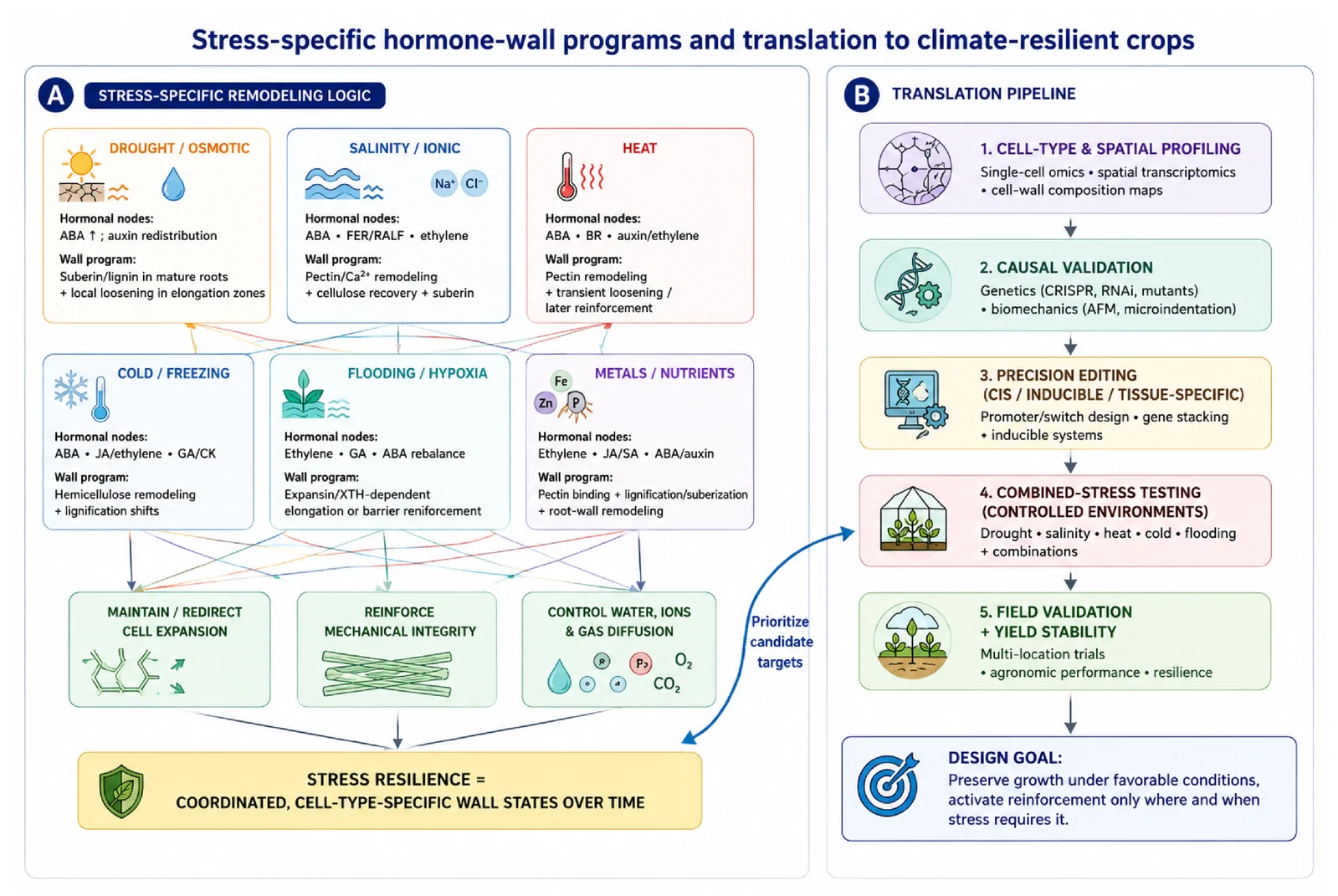
Stress-specific hormone–cell-wall remodeling and its translation into climate-resilient crops. (**A**) Abiotic stresses activate distinct but interconnected hormonal networks that regulate cell-wall remodeling, including pectin and cellulose modification, expansin/XTH-mediated wall dynamics, and lignin/suberin deposition. These responses converge on the control of cell expansion, mechanical reinforcement, and water, ion, and gas transport, generating coordinated, cell-type-specific wall states that support stress resilience. (**B**) Translational pipeline linking cell-type and spatial profiling with causal validation, precision editing, combined-stress testing, and field evaluation. Candidate regulatory targets are progressively prioritized to develop crops that maintain growth under favorable conditions while activating stress-responsive wall reinforcement when and where required.

Sequential and fluctuating stresses require different interpretation. In a sequential design, the first stress can alter hormone sensitivity, wall composition, anatomy, or chromatin state before the second stress begins, so A-B cannot be assumed to be equivalent to B-A. Fluctuating stresses repeatedly move tissues between different stress intensities and may favor reversible wall remodeling rather than stable reinforcement. Recovery is also an active phase rather than a simple return to an unstressed baseline. After short-term water deficit, sunflower and tomato showed time-dependent changes in cellulose, hemicellulose, pectin, and lignin during 3–24 h of rewatering, together with progressive recovery of photosynthetic conductance, directly demonstrating that wall composition continues to change after stress removal [118]. However, these compositional changes do not by themselves establish corresponding changes in wall mechanics. Ultimately, resolving how cell-wall plasticity integrates with hormonal crosstalk and stress memory will be essential for developing crops with durable resilience to the complex and sequential stresses encountered under field conditions. The stress-specific hormone–wall programs discussed above and their translational integration are illustrated in Figure 3. Stress memory should be reserved for persistent effects of prior exposure that modify a later response, rather than for incomplete recovery alone. Priming and memory can involve hormone sensitivity, chromatin accessibility, DNA methylation, histone modifications, transcriptional competence, metabolism, and anatomy [98,99]. A wall-associated example comes from Arabidopsis root suspension cells adapted to high salinity: adapted cells developed thicker walls with higher lignin content, while acquired salt tolerance persisted after stress relief; importantly, the study did not demonstrate that the altered wall state itself was the causal carrier of memory [119]. Therefore, scenarios such as drought-induced suberization altering a later salinity response should be presented as mechanistically plausible hypotheses unless the sequential experiment directly measures retention of the wall state and its contribution to the second-stress phenotype. The sunflower and tomato studies reflect recovery at the organ or whole-plant level, while the Arabidopsis root suspension-cell study provides evidence of stress memory at the cellular level rather than direct proof of a whole-plant mechanism.

Future studies should therefore separate these temporal categories experimentally rather than pooling them under the term combined stress. A rigorous design should include each single stress, simultaneous A+B treatment, both sequential orders (A-B and B-A), defined recovery intervals, and, where relevant, repeated or fluctuating cycles. Time-resolved measurements should connect hormone and CWI signaling with wall chemistry and architecture, direct mechanical or transport measurements, and physiological or whole-plant outcomes. This framework will distinguish true cross-tolerance or stress memory from transient carry-over, incomplete recovery, or additive damage, and will clarify which wall responses are directly demonstrated and which remain extrapolated from single-stress studies [97,98,99]. Overall, clearly separating these different stress patterns helps explain whether cell-wall remodeling is a rapid response to stress, part of the recovery process, or a longer-lasting adaptive change that influences how plants respond to future stress.

### 6.8. Critical Comparative Synthesis and Analytical Prospects

Comparative evidence across abiotic stresses indicates that no single cell-wall phenotype can be universally associated with stress tolerance. Drought generally favors hydraulic conservation and barrier formation while preserving localized extensibility; salinity introduces additional demands related to ion binding, selective exclusion, and maintenance of wall integrity; heat can involve rapid and potentially reversible pectin and guard-cell remodeling followed by structural recovery or reinforcement; cold and freezing are associated with adjustments in wall compliance and rigidity that help tissues accommodate cellular dehydration and extracellular ice while maintaining integrity; flooding can favor either extensive wall loosening during escape growth or growth restraint during quiescence; and heavy-metal or nutrient-associated stresses often place greater emphasis on wall chemistry in ion binding, sequestration, and buffering [14,38,41,93,104,109,110]. These contrasts illustrate why increased lignification, suberization, pectin cross-linking, or wall stiffness can be beneficial in one tissue or stress phase yet detrimental in another. The relevant adaptive variable is therefore not simply the abundance of an individual wall polymer, but the timing, location, reversibility, and physiological cost of the resulting wall state. The strength of mechanistic evidence also differs among stress classes. Salinity provides well-resolved examples of CWI signaling through FER-dependent Ca^2+^ signaling and LRX–RALF–FER modules [59,68,69], whereas drought includes defined ABA-linked pathways such as ABA–SnRK2–NST1 regulation of secondary-wall formation and ABA-responsive control of root suberization [13,42]. Flooding provides direct evidence linking ethylene signaling with expansin-mediated wall loosening during adaptive elongation [14], while cadmium stress provides a mechanistic connection between EIN3 signaling and XTH33-mediated wall remodeling [88]. By contrast, much of the evidence for cold, nutrient imbalance, broader heavy-metal responses, and combined stresses remains based on transcriptional, compositional, or whole-plant physiological associations rather than experiments that directly connect hormone activity, wall remodeling, mechanical change, and improved stress performance.

For cross-stress comparison, evidence should therefore be evaluated at matched levels: molecular or regulatory, biochemical or structural, mechanical or transport, physiological, and agronomic. Changes in CESA-, PME-, XTH-, expansin-, lignin-, or suberin-related transcripts should not be treated as equivalent to demonstrated changes in stiffness, extensibility, permeability, or hydraulic function, and measured mechanical changes should not automatically be interpreted as improved whole-plant performance. This framework helps distinguish complete causal chains from cases in which intermediate steps remain inferred. Future studies should adopt a common comparative framework across abiotic stresses rather than treating each stress as an isolated catalogue of wall responses. Within comparable tissues and developmental zones, time-resolved experiments should integrate measurements of hormone abundance or signaling activity, apoplastic pH and Ca^2+^ dynamics, wall-polymer composition, pectin methylesterification, lignin and suberin deposition, wall stiffness or extensibility, water or ion transport, and growth or yield consequences. Factorial and sequential designs will be particularly important for determining whether wall responses are stress-specific, shared, antagonistic, or involved in cross-tolerance and stress memory [97,98,99,117]. Translation to crops should additionally account for structural differences between eudicot and grass cell walls and should prioritize inducible and tissue-specific remodeling strategies that preserve resource acquisition and growth under favorable conditions. From this perspective, the most promising targets are likely to be those that enable rapid and spatially controlled transitions between permissive and protective wall states while minimizing penalties to growth and yield.

## 7. Molecular Control of Hormone-Dependent Cell-Wall Remodeling

### 7.1. Transcriptional Regulators: NAC, MYB, WRKY, ERF, bZIP, ARF, and Related Factors

Hormonal and CWI signals ultimately influence wall structure by changing transcription of biosynthetic enzymes, transporters, remodeling proteins, and regulatory factors. Several transcription-factor families repeatedly appear at this interface. NAC and MYB proteins are particularly important for secondary-wall biosynthesis, whereas ARFs, ERFs, bZIPs, WRKYs, and other factors translate auxin, ethylene, ABA, JA/SA, and stress signals into cell-type-specific gene expression. The NAC-MYB hierarchy controlling secondary-wall formation is one of the best-established examples. NAC factors such as NST1 activate downstream MYB regulators and structural genes associated with cellulose, hemicellulose, and lignin production. ABA can directly regulate this system through SnRK2-dependent phosphorylation of NST1, demonstrating how a stress hormone can act on a core wall transcription factor rather than merely changing upstream physiology [13]. Broader reviews of NAC proteins show that these factors integrate ABA, ethylene, ROS, and MAPK signals across drought, salinity, and temperature stress [120,121]. MYB proteins also control root barrier formation. MYB41, MYB53, MYB92, and MYB93 regulate suberin biosynthesis and environmental plasticity in Arabidopsis, while MYB92-related functions have been co-opted into the tomato exodermal suberin program [42,43]. These examples show how conserved transcriptional modules can be redeployed in different cell layers across species.

ARFs provide a direct route from auxin perception to wall remodeling. Auxin-regulated transcription influences pectin-remodeling genes, expansins, and other growth-associated factors, while rapid TMK-dependent signaling acts in parallel at the plasma membrane. Ethylene-responsive EIN3/EIL and ERF proteins similarly regulate stress-dependent growth programs; EIN3 control of XTH33 under cadmium stress is a clear example [88]. ERF-VII proteins dominate low-oxygen transcription and thereby influence the developmental programs that require wall remodeling during flooding. WRKY and bZIP factors contribute to ABA, ROS, SA/JA, and stress signaling and can influence lignin, phenylpropanoid, and wall-defense pathways. Their roles are often pleiotropic, making it difficult to attribute stress tolerance specifically to wall remodeling. The most informative future studies will therefore connect transcription-factor perturbation to biochemical wall phenotypes, mechanical measurements, and tissue-specific expression rather than relying on survival or biomass alone. Accordingly, transcription-factor evidence should be interpreted according to how far it progresses along this causal chain. For example, ABA-SnRK2-NST1 and EIN3-XTH33 provide mechanistic links between upstream signaling and wall-related regulatory outputs [13,88], but direct biomechanical measurements are still required before concluding that these pathways alter wall stiffness, extensibility, or related mechanical properties.

### 7.2. Post-Transcriptional and Epigenetic Regulation

Transcriptional networks are further shaped by RNA processing, non-coding RNAs, protein turnover, chromatin state, and DNA methylation. These layers are important because wall remodeling is temporally demanding: some responses must occur within minutes, whereas others need to persist through days of stress or remain primed for recurring stress. MicroRNAs can target transcription factors and wall-related genes involved in lignification, pectin metabolism, and metal fixation. In heavy-metal stress, recent synthesis highlights miRNA-mediated regulation of genes controlling wall biosynthesis and modification [93]. Small RNAs can therefore connect stress perception with quantitative adjustment of structural pathways. Epigenetic regulation provides a mechanism for longer-term plasticity. DNA methylation, histone modifications, histone variants, chromatin remodeling, and RNA modifications all contribute to abiotic-stress responses [99]. Some stress-induced chromatin states persist after stress removal and can modify the response to later exposure, creating physiological memory. Although wall-specific epigenetic mechanisms remain less comprehensively mapped than general stress pathways, lignin, cellulose, hormone, and root-development genes are plausible targets because they undergo extensive transcriptional reprogramming during acclimation. The emerging possibility of targeted epigenome editing is especially relevant for wall engineering. Instead of permanently knocking out a pleiotropic structural gene, CRISPR/dCas-based activators, repressors, or epigenetic modifiers could alter expression in a tissue- or environment-dependent manner. Such approaches remain largely prospective for hormone-wall traits but align well with the need to avoid constitutive growth penalties [122,123]. Together, these regulatory layers position the cell wall as a dynamic target of stress memory, opening the possibility of engineering plants that remodel their walls only when and where needed, thereby improving resilience without compromising normal growth. Similarly, miRNA- or epigenetic-associated changes should be treated as regulatory evidence until effects on target protein or enzyme activity and the corresponding wall chemistry are demonstrated. Transcriptomic or epigenomic changes alone should therefore not be equated with altered wall mechanics or improved stress tolerance.

### 7.3. Spatial and Temporal Control of Wall Remodeling

The biological meaning of wall remodeling depends on where and when it occurs. Whole-organ RNA-seq can obscure this because opposite responses in different cell types may average to a modest net change. A root elongation zone, mature endodermis, xylem vessel, guard cell, and root hair have different wall compositions, hormone environments, mechanical functions, and stress priorities. Therefore, hormone-wall regulation must be resolved spatially. Time is equally important. Early stress responses can include pH shifts, Ca^2+^ signals, ROS, receptor phosphorylation, and rapid changes in enzyme activity; later responses involve transcription, wall biosynthesis, lignification, suberization, and developmental reprogramming. A single end-point measurement cannot distinguish early adaptive loosening from later reinforcement. This problem is particularly acute under heat and combined stress, where stress phases can reverse the direction of wall change [104]. Single-cell and single-nucleus transcriptomics now allow cell-type-specific stress responses to be resolved at much higher resolution. Recent analyses show that these approaches can identify stress-responsive cell populations and candidate genes that are invisible in bulk measurements [124,125]. However, protoplasting can itself trigger stress responses and preferentially lose recalcitrant cell types, so single-nucleus methods and spatial validation are increasingly important. Therefore, spatial and temporal transcriptomic data should ideally be paired with protein or enzyme measurements, direct analysis of wall chemistry or architecture, and mechanical or transport phenotyping in the same tissue and time window. This integrated approach is necessary to connect regulatory changes to downstream wall function rather than interpreting transcript abundance in isolation.

### 7.4. Tissue and Cell-Type-Specific Responses

Tissue specificity is not a technical detail but a core feature of wall adaptation. Guard cells require flexible pectin-rich walls that convert turgor into stomatal movement; xylem requires lignified secondary walls; root endodermal and exodermal cells construct diffusion barriers; epidermal cells interact directly with soil or air; and elongating cells require controlled yielding. A stress response that benefits one cell type may be harmful in another. The tomato exodermis illustrates this principle. Arabidopsis lacks an exodermal layer comparable to many crops, yet exodermal suberin is functionally important for tomato drought tolerance [43]. Similarly, cereal walls differ from eudicot walls in hemicellulose composition and mixed-linkage glucans. Mechanisms established in Arabidopsis should therefore be treated as hypotheses for crops rather than universal templates. Spatial transcriptomics and spatial multi-omics preserve positional information and can map wall-related gene expression within intact tissue architecture. Recent reviews emphasize their potential for drought, salinity, mechanical stress, and crop improvement, while noting challenges created by rigid walls, autofluorescence, tissue sectioning, and computational integration [124,126,127]. These tools should make it possible to identify low-pleiotropy targets whose manipulation is restricted to the cell layers most important for resilience.

## 8. From Mechanism to Climate-Resilient Crops

### 8.1. Genetic Targets for Engineering Hormone-Responsive Cell Walls

The most attractive engineering targets are not necessarily the genes with the largest stress-induced expression changes. Useful targets should have a mechanistically validated effect on wall properties, a favorable trade-off between stress protection and growth, and a degree of spatial or temporal specificity that minimizes pleiotropy. Several classes meet these criteria. CWI receptors such as FER and THE1 are appealing because they regulate wall-state sensing and hormone feedback rather than a single structural polymer. Yet receptor manipulation carries risk because FER affects reproduction, growth, immunity, and multiple hormone pathways. Precision engineering of ligand interactions, tissue-specific expression, or downstream branches may therefore be preferable to constitutive receptor knockout or overexpression [9,58].

Wall-modifying proteins are another target class. Expansins and XTHs could preserve root growth under moderate drought or salinity, but constitutive loosening may weaken tissues. PMEs and PME inhibitors offer control over pectin charge and mechanics, but isoform-specific functions and Ca^2+^ context are critical. Laccases, peroxidases, NAC/MYB regulators, and suberin regulators can reinforce structural or barrier tissues, yet excessive activation can reduce growth. The translational challenge is therefore to tune where and when these genes act. Root barriers offer particularly promising targets because they can alter water and ion flux with limited effects on aerial growth. The genetic demonstration that exodermal suberin supports tomato drought tolerance and that ABA-responsive MYBs control endodermal suberization provides a rational basis for engineering stress-responsive barriers [42,43]. Similar approaches could be tested for salt exclusion, nutrient efficiency, and flooding-related gas barriers.

### 8.2. Genome Editing and Precision Breeding

CRISPR-Cas technology now provides a versatile toolbox for precision crop improvement, including conventional nuclease editing, base editing, prime editing, multiplex editing, transcriptional regulation, and emerging epigenome-engineering approaches [122]. These technologies can generate loss-of-function alleles, introduce precise nucleotide substitutions, modify protein domains, and alter promoters or other cis-regulatory elements without necessarily disrupting coding sequences. For hormone-responsive cell-wall traits, regulatory-element editing may be particularly valuable because many genes controlling cellulose, lignin, pectin, suberin, and wall-associated signaling are pleiotropic. Complete disruption of such genes may improve stress tolerance but simultaneously impair growth, vascular development, fertility, or yield. Editing stress-responsive promoter elements offers a more refined strategy in which gene activity could be modified predominantly under drought, salinity, heat, or flooding while normal developmental expression is retained [122]. The feasibility of this concept has recently been demonstrated in crops: precise insertion of a heat-shock element into promoters of cell-wall-invertase genes generated heat-responsive expression and improved yield resilience in rice and tomato under both controlled and field conditions [128]. However, epistatic and compensatory responses should be tested empirically because multiplex perturbation can also generate unanticipated pleiotropy.

Precision breeding should also exploit natural genetic diversity rather than relying exclusively on newly designed mutations. Pangenomes, GWAS, haplotype analyses, and multi-omics approaches can reveal favorable alleles and regulatory variants that are absent from a single reference genome and identify candidate loci for precise editing [129,130]. For cell-wall-mediated stress tolerance, this approach could identify natural variation affecting pectin methylesterification, lignin composition, suberin deposition, receptor sensitivity, hormone transport, or root-barrier formation. Once validated, favorable alleles could be recreated, fine-tuned, or combined in elite cultivars through genome editing. Multiplex strategies may be particularly useful for complex stress traits because several components of a hormone–wall network can be modified simultaneously, potentially avoiding strong phenotypic effects caused by manipulating a single major regulator [122,131].

The major challenge, however, is translating successful editing from controlled experiments into agronomically useful resilience. Survival of acute laboratory stress does not necessarily predict stable performance under fluctuating field conditions. Edited wall traits therefore need to be evaluated not only for stress survival but also for yield, hydraulic conductance, nutrient acquisition, root architecture, lodging resistance, disease susceptibility, reproductive performance, and grain or fruit quality. Recent assessments of crop stress editing emphasize that field validation and genotype × environment testing remain essential even when promising stress-tolerance phenotypes are obtained under controlled conditions [131]. Accordingly, the greatest value of precision breeding for hormone-cell-wall engineering will come from moving beyond simple gene knockout toward allele-, dosage-, tissue-, and environment-specific regulation, thereby enhancing stress resilience without sacrificing the growth and productivity required under favorable conditions.

### 8.3. Multi-Omics and Spatial Technologies for Target Discovery

Multi-omics can connect genomic variation with hormone levels, transcription, protein abundance, metabolites, wall composition, and phenotype. This is particularly useful for wall biology because many key processes are poorly inferred from transcript abundance alone. Proteomics can reveal enzyme abundance and phosphorylation; metabolomics can detect phenylpropanoid or hormone changes; glycomics can characterize wall polymers; and ionomics can quantify nutrient or toxic-metal accumulation. Single-cell and spatial methods add the missing positional dimension. scRNA-seq and snRNA-seq reveal cell-type-specific transcriptional states, while spatial transcriptomics maps expression back into intact tissue. Recent reviews show rapid progress in Arabidopsis, maize, soybean, and other plants, including stress-responsive cell types and developmental trajectories [124,125]. Spatial omics is increasingly being positioned as a route from gene discovery to crop improvement because it can identify the exact cell layers in which a target operates [126].

The next step is spatial multi-omics. Integrating transcriptomics with chromatin accessibility, proteins, metabolites, or cell-wall chemistry could distinguish a transcriptionally activated wall gene from a functionally remodeled wall domain. A recent synthesis highlights the rapid expansion of scRNA-seq, snATAC-seq, spatial transcriptomics, and spatial metabolomics, while emphasizing data integration, batch effects, reference quality, and the need for plant-specific computational resources [127]. For hormone-wall research, an ideal experiment would combine cell-type-resolved hormone reporters, spatial transcriptomics, wall glycomics or imaging, and mechanical measurements across a stress time course. Such datasets could identify causal modules rather than simple correlations and would provide a strong basis for selecting low-pleiotropy breeding targets.

### 8.4. Cell-Wall Phenotyping and Mechanobiology

Genetic and omics studies need to be complemented by direct measurements of the cell-wall phenotype. Traditional biochemical assays can quantify cellulose, lignin, monosaccharides, or the degree of pectin esterification, but they provide limited information about spatial organization, local mechanics, and dynamic wall behavior. Modern mechanobiology therefore adds techniques such as atomic force microscopy (AFM), nanoindentation, extensometry, Brillouin microscopy, confocal imaging, Raman and infrared spectroscopy, solid-state NMR, and live reporters of pH, Ca^2+^, ROS, or receptor activity. Among these approaches, AFM is particularly valuable because it can resolve nanoscale wall topography, mechanical properties, molecular interactions, and surface characteristics under near-physiological conditions. Recent advances also allow AFM to be combined with molecular-recognition measurements, Kelvin-probe approaches, and infrared or Raman spectroscopy, providing simultaneous structural, mechanical, and chemical information about the plant cell wall [53]. These approaches are important because they can distinguish whether a genetic, hormonal, or environmental treatment directly alters wall stiffness, surface organization, molecular interactions, or chemical composition rather than relying only on indirect measurements of growth. However, mechanobiological data must be interpreted together with tissue hydration and turgor pressure, because the apparent stiffness of living tissues reflects both intrinsic wall material properties and mechanical forces generated by pressurized cells [54]. This distinction is especially important under drought and salinity, where rapid changes in water status and turgor can strongly influence mechanical measurements. Spatial resolution is equally critical because wall properties differ among cell types, developmental zones, and individual wall domains; averaging measurements across an entire root or leaf may therefore obscure the local heterogeneity that enables stress adaptation. A major remaining challenge is throughput. AFM and advanced spectroscopic methods provide detailed mechanistic information but are relatively slow compared with field-scale phenotyping, whereas remote sensing and imaging platforms generally measure organ- or canopy-level outcomes rather than wall properties directly. A translational phenotyping pipeline will therefore require careful calibration between mechanistic laboratory measurements and scalable proxies such as growth dynamics, hydraulic traits, spectral signatures, tissue deformation, or imaging-derived features. Integrating these approaches will be essential for linking gene function and hormone signaling to measurable wall mechanics and, ultimately, to crop performance under environmental stress.

### 8.5. Translating Laboratory Mechanisms to Field Performance and Yield Stability

A major challenge in hormone-cell-wall research is translating molecular and seedling-level phenotypes into stable agronomic performance. Much of the current mechanistic knowledge is derived from seedlings exposed to short-term or relatively severe stresses under hydroponic, agar-based, greenhouse, or other controlled conditions. Field-grown crops, however, experience fluctuating stress intensity, heterogeneous soils, variable nutrient and water availability, microbial interactions, competition, and repeated stress–recovery cycles across different developmental stages. Consequently, stress-tolerance mechanisms that perform well under controlled conditions may provide little benefit, or even reduce productivity, when transfer to realistic agricultural environments [132,133]. Recent analyses therefore advocate a “field-to-lab-to-field” framework in which environmental conditions relevant to production systems are incorporated into mechanistic experiments and promising traits are returned to multi-environment trials for validation. For cell-wall traits, such validation is particularly important because structural adaptations frequently involve trade-offs between stress protection and growth. Increased lignification can strengthen tissues, maintain vascular integrity, and improve resistance to environmental stress, but excessive or poorly targeted changes in lignin biosynthesis can alter hydraulic function, defense signaling, growth, and biomass accumulation [39,134]. Similarly, enhanced root suberization can reduce uncontrolled water loss and restrict the entry of potentially toxic ions, but root diffusion barriers also regulate water and nutrient movement between soil and vascular tissues; therefore, constitutive reinforcement may impose costs under favorable or nutrient-limited conditions [50]. Sustained wall loosening may help maintain growth during stress yet reduce mechanical strength, while changes in hormone sensitivity may have additional consequences for flowering, senescence, immunity, root architecture, and reproductive development. These examples emphasize that cell-wall modification should not be evaluated solely by survival after an acute stress treatment; the associated costs to growth and productivity must be quantified across the complete crop life cycle. Genotype and environment interaction is therefore central to translating wall biology into crop improvement. A wall phenotype advantageous in saline or drought-prone soil may be neutral or detrimental under well-watered, nutrient-rich conditions. Stress-inducible, tissue-specific, or developmentally restricted modifications are thus likely to be more useful than constitutive changes because they can provide protection while minimizing penalties when stress is absent. Multi-location and multi-season experiments should evaluate not only average yield but also yield stability, water- and nutrient-use efficiency, hydraulic performance, root architecture, lodging resistance, reproductive success, product quality, and recovery after stress [132,133]. More importantly, successful translation of hormone–cell-wall mechanisms will depend not on maximizing stress resistance in isolation, but on identifying wall traits that maintain a productive balance between protection, resource acquisition, growth, and yield across variable field environments.

## 9. Knowledge Gaps and Future Research Priorities

Despite substantial progress in understanding phytohormone signaling and cell-wall remodeling under abiotic stress, the field remains limited by fragmentation across molecular, mechanical, developmental, and agronomic scales. Many studies identify changes in hormone levels, wall-related transcripts, or polymer abundance, but comparatively few establish a complete causal sequence linking stress perception to wall modification and, ultimately, to physiological performance. A major priority is therefore to connect environmental perturbation with cell-wall/CWI perception, hormone and second-messenger signaling, specific wall-remodeling machinery, measurable mechanical or transport changes, and whole-plant stress adaptation. Achieving this level of integration will require stronger causal experiments, improved spatial and temporal resolution, realistic stress regimes, quantitative mechanobiology, and validation in crops.

### 9.1. Moving from Correlation to Causal Hormone-Wall Mechanisms

A persistent limitation is the tendency to infer mechanism from parallel changes in hormone concentrations and wall-related gene expression. Such associations are useful for identifying candidates but do not demonstrate that a hormone directly causes a particular wall phenotype. Future studies should distinguish at least three levels of evidence: association, functional requirement, and direct mechanism. Transcriptomic or hormonal correlations establish association; genetic perturbation can demonstrate functional requirement; and biochemical interaction, temporal ordering, rescue experiments, or biophysical measurements are needed to establish direct causality. Mechanistically strong examples already include FERONIA-dependent Ca^2+^ signaling during salt-induced wall perturbation, TMK-mediated activation of H^+^-ATPases during auxin-induced wall acidification, ABA-SnRK2 regulation of NST1-dependent secondary-wall formation, and the genetic demonstration that exodermal suberin contributes to tomato drought tolerance [11,13,43,59]. Future work should increasingly combine inducible genetic perturbation with direct wall chemistry and mechanical phenotyping so that changes in transcription can be linked to specific structural functions.

### 9.2. Resolving Cell-, Tissue-, and Developmental-Stage Specificity

Hormone-wall regulation is fundamentally spatial. Guard cells, elongating root epidermal cells, root hairs, endodermal and exodermal cells, xylem vessels, and reproductive tissues differ greatly in wall composition, mechanical function, hormone environment, and stress sensitivity. Consequently, the same wall enzyme or hormone may produce beneficial effects in one cell type but detrimental effects in another. Whole-organ measurements can obscure these opposing responses. Single-cell and single-nucleus transcriptomics, spatial transcriptomics, lineage-specific reporters, and spatial multi-omics now provide opportunities to resolve these differences and identify low-pleiotropy targets for crop improvement [125,126]. However, transcriptional resolution alone is insufficient; spatial gene-expression maps should be integrated with localized measurements of wall composition, pH, stiffness, ion transport, and hormone activity. Comparative studies across crop species are also essential because grass wall architecture, exodermal development, polyploidy, and vascular organization differ substantially from the Arabidopsis systems on which many current models are based.

### 9.3. Understanding Combined, Sequential, and Fluctuating Stresses

Most mechanistic studies still examine one stress at a fixed intensity, whereas agricultural environments are characterized by combinations and temporal sequences of stresses. Drought and heat may occur simultaneously, salinity can interact with nutrient deficiency, and flooding is frequently followed by rapid reoxygenation. Such combinations often generate non-additive physiological and transcriptional responses and may impose conflicting demands on wall mechanics [97]. Sequential stresses are equally important because an initial stress can modify root barriers, wall composition, hormone sensitivity, or chromatin state before a second stress occurs. Stress memory may therefore change the mechanical and biochemical starting state of the wall during subsequent exposure [98,99]. Future experiments should move beyond single end-point comparisons toward time-resolved factorial and sequential designs that include stress onset, acclimation, recovery, and repeated exposure. Direct measurements of pectin status, lignification, suberization, wall mechanics, and hydraulic behavior should be integrated with hormone profiling and multi-omics to determine whether altered wall states actually contribute to cross-tolerance.

### 9.4. Integrating Hormone Signaling with Cell-Wall Mechanobiology

Another major gap lies between molecular signaling studies and quantitative analysis of cell-wall mechanics. Hormone studies frequently measure gene expression, signaling activity, or growth, whereas mechanobiological studies quantify properties such as stiffness, strain, deformation, extensibility, or wall architecture. These datasets are rarely integrated sufficiently to establish how a defined hormone-induced biochemical modification changes the physical properties experienced by the cell. Future studies should therefore combine measurements of hormone dynamics, wall composition, apoplastic pH, Ca^2+^ and ROS signaling, turgor pressure, wall strain, and CWI receptor activity within the same experimental framework. AFM can provide nanoscale information on wall topography and apparent mechanical properties, while extensometry, creep analysis, strain mapping, Brillouin microscopy, Raman spectroscopy, and advanced live-cell imaging can provide complementary mechanical, structural, and chemical information [53]. Importantly, measurements of apparent tissue stiffness should be interpreted together with hydration, geometry, and turgor because the mechanical behavior of living tissues reflects contributions from both wall properties and cellular pressure [54]. Establishing quantitative relationships between defined mechanical perturbations and activation of FER, THE1, WAK, MIK2, or related signaling pathways would represent an important step toward a predictive model of CWI–hormone regulation. A practical evidentiary standard follows from this distinction. Studies claiming that a hormone, receptor, transcription factor, or wall-remodeling gene alters wall mechanics should ideally connect perturbation of the regulator to: (i) a molecular or signaling response; (ii) a corresponding biochemical or structural wall change; (iii) a direct mechanical or transport measurement; and (iv) a physiological outcome in the same tissue and within an appropriate temporal window. Mechanical readouts may include AFM-derived modulus, extensometry or creep analysis, strain mapping, and other validated measurements of deformation or extensibility, whereas transport-related claims require independent measurements of hydraulic conductance, solute permeability, or ion flux. Failure to measure every intermediate step does not invalidate a biological observation, but it should limit the mechanistic interpretation to the level directly supported by the available evidence [53,54].

### 9.5. Linking Molecular Wall Remodeling to Crop Yield and Resilience

The translational value of a wall mechanism ultimately depends on whether it improves crop performance without creating unacceptable costs. Many candidate genes increase survival under controlled stress, but relatively few wall traits have been connected to stable productivity across environments. This problem is particularly important because increased lignification, suberization, wall stiffening, or prolonged wall loosening can generate trade-offs involving growth, hydraulic conductance, nutrient uptake, pathogen susceptibility, lodging, reproductive development, and carbon allocation. Crop studies should therefore treat yield stability and recovery capacity as mechanistic outcomes, rather than as secondary measurements made after stress-tolerance screening. Multi-location and multi-season experiments should connect the engineered wall phenotype with root water uptake, ion exclusion, canopy temperature, reproductive success, grain or fruit development, and post-stress recovery [132,133]. Genotype and environment interactions must also be explicitly quantified because a wall state advantageous during drought or salinity may become neutral or costly under favorable conditions. This strengthens the case for stress-inducible and tissue-specific regulation rather than constitutive wall modification.

### 9.6. Toward Predictive and Design-Based Cell-Wall Engineering

**Figure 4:**
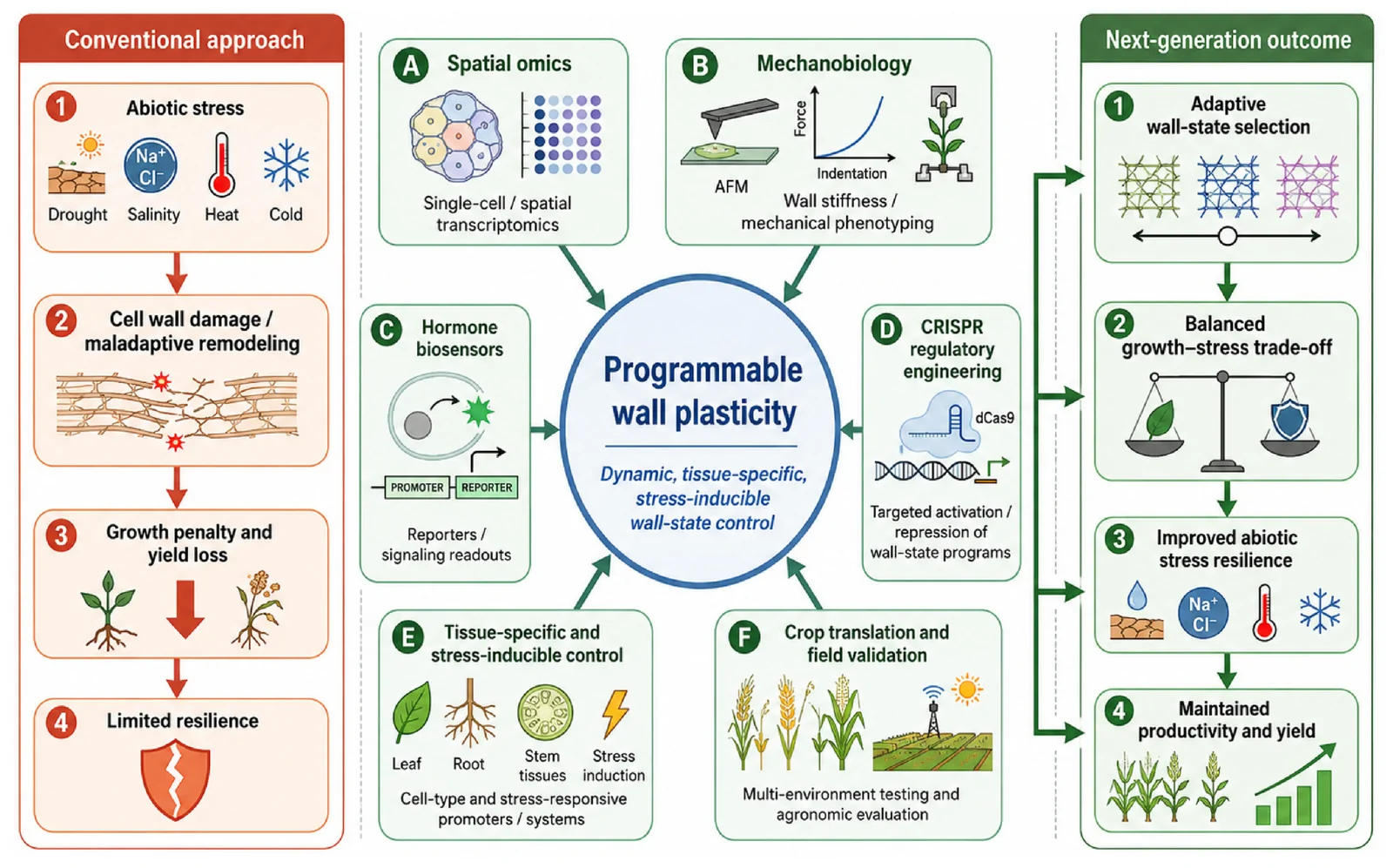
Future crop engineering roadmap for programmable wall plasticity. Conventional approaches to stress improvement often emphasize generalized wall reinforcement, which may enhance protection but can negatively affect growth and resource allocation. A future strategy is to engineer programmable wall plasticity through the integration of spatial omics, mechanobiology, hormone biosensors, CRISPR-based regulatory engineering, tissue-specific and stress-inducible control systems, and field-level validation. These approaches aim to enable adaptive wall-state selection, optimize growth–stress trade-offs, improve abiotic-stress resilience, and maintain productivity. The framework highlights a shift from constitutively stronger walls toward dynamic, context-dependent regulation of wall properties.

The longer-term goal should be to move from descriptive stress biology toward predictive design of adaptive wall states. Such a framework would integrate regulatory variation, hormone signaling, CWI perception, polymer chemistry, wall mechanics, hydraulic behavior, and whole-plant growth to predict how a targeted genetic change will affect stress performance. Spatial multi-omics and quantitative mechanobiology can identify where and when a candidate pathway functions, while CRISPR-based regulatory editing can test whether its activity can be tuned without disrupting normal development [122]. Particularly promising strategies include promoter and cis-regulatory editing, tissue-specific regulation, stress-inducible expression, and multiplex modification of interacting pathway components. Recent demonstrations that regulatory editing can improve stress-responsive crop performance illustrate the potential of moving beyond simple knockout strategies [128]. Nevertheless, predictive models will require standardized phenotyping, comparable environmental treatments, cross-species validation, and sufficiently large field datasets before they can reliably guide breeding decisions. Taken together, these priorities define a transition from cataloguing stress-responsive wall genes toward a causal, spatially resolved, mechanically quantitative, and agronomically validated framework for programmable wall plasticity. The central future challenge is not simply to identify mechanisms that strengthen or loosen the wall, but to determine how plants can generate the appropriate wall state in the appropriate cell, at the appropriate time, and with minimal cost to growth and yield. These research priorities are synthesized into a roadmap for programmable wall plasticity in Figure 4.

## 10. Conclusions

The plant cell wall is now understood as an active and highly responsive part of plant adaptation to abiotic stress. Beyond providing structural support, it is continuously remodeled to adjust growth, water relations, ion transport, and tissue protection as environmental conditions change. These responses involve coordinated changes in cellulose, pectin, lignin, suberin, and wall-associated proteins, together with cell-wall integrity signaling mediated by FERONIA, THESEUS1, MIK2, WAKs, RALF peptides, Ca^2+^, ROS, and MAPK pathways. Through their interaction with phytohormone signaling, these components help plants sense changes in wall status and adjust growth and defense accordingly. A key message emerging from this review is that stress tolerance is not achieved simply by making cell walls stronger or more flexible. Instead, successful adaptation depends on the ability to modify wall properties in the right tissue, at the right stage, and for the appropriate duration. Future work should therefore focus on linking well-defined molecular mechanisms with spatial wall behavior, mechanical properties, and whole-plant performance. Combining causal genetics, spatial multi-omics, mechanobiology, precision genome editing, and field-based validation will be especially important for identifying wall traits that improve resilience without imposing penalties on growth, nutrient uptake, reproduction, or yield. In this way, a better understanding of hormone-regulated wall plasticity could provide a practical foundation for developing crops that remain productive under increasingly variable and challenging environments.

## Figures and Tables

**Table 1:** Major plant cell-wall remodeling modules in stress and hormone signaling.

Wall Module	Core Machinery	Main Wall Effect	Stress/Hormone Links	References
Cellulose biosynthesis and organization	CESA-containing CSCs; cortical microtubules; CSI1/CC proteins; trafficking regulators	Builds the load-bearing cellulose network; controls strength, microfibril orientation, anisotropy, and directional growth.	Salt, osmotic, temperature, and mechanical stresses alter CSC dynamics and microtubules. CESA phosphorylation and BR/auxin pathways regulate cellulose deposition.	[3,32,44,45]
Xyloglucan/hemicellulose remodeling	XTHs; XET and XEH activities	Cleaves or relegates xyloglucan, restructuring cellulose-associated networks and wall extensibility.	Responsive to drought, salinity, cold, osmotic stress, metals, flooding, and hormones; may promote loosening or reinforcement depending on context.	[46,47]
Pectin methyl esterification	PMEs; PMEIs	Controls homogalacturonan de-methyl esterification, affecting Ca^2+^ cross-linking, stiffness, porosity, adhesion, and enzyme susceptibility.	Integrated with CWI, auxin, ABA, BR, and stress responses; PME/PMEI balance shifts with salinity, drought, temperature, and biotic stress.	[21,48]
Pectin depolymerization	PGs; pectate/pectin lyases; PG inhibitors	Shortens pectin chains, altering adhesion, porosity, and mechanics; controlled cleavage releases signaling OGs.	Links developmental remodeling with wound/defense signaling; OGs activate CWI/defense pathways and influence hormone-regulated growth.	[21,48]
Expansin-mediated wall loosening	α-expansins; β-expansins; H^+^-ATPase-driven acidification	Promotes wall yielding by disrupting noncovalent interactions without polysaccharide hydrolysis.	Central to auxin-driven acid growth and adaptive responses to flooding, drought, salinity, and related stresses.	[34]
Lignification/oxidative reinforcement	Monolignol pathway; laccases; class III peroxidases	Increases wall rigidity, hydrophobicity, vascular strength, and resistance to deformation or penetration.	Often induced by drought, salinity, metals, wounding, and pathogens through ABA- and ROS-linked signaling; may reduce growth plasticity.	[23,49]
Suberization/apoplastic barriers	ABA-responsive MYBs; GPAT/CYP86/FAR enzymes; ABCG transporters	Forms hydrophobic barriers that restrict apoplastic water and solute movement and regulate root radial transport.	Strongly induced by drought and salinity; ABA-dependent programs adjust suberin amount and distribution to limit water loss and ion entry.	[42,50]
ROS-dependent wall chemistry	RBOHs; class III peroxidases; apoplastic redox systems	ROS can stiffen walls through oxidative cross-linking or promote loosening through radical-mediated reactions; also affects lignification.	Couples CWI, Ca^2+^, hormone, and stress signaling; ROS balance changes under drought, salinity, temperature extremes, metals, and pathogens.	[5,49]
CWI sensing/mechanochemical signaling	WAK/WAKLs; FERONIA/THESEUS1; RALF-LRX modules; Ca^2+^/ROS signaling	Senses wall mechanics, pectin status, and damage, triggering compensatory remodeling, growth, and defense.	Connects wall perturbation to Ca^2+^, ROS, MAPK, and ABA/BR/ethylene/jasmonate pathways during abiotic and biotic stress.	[48,51,52]

Abbreviations: ABA, abscisic acid; BR, brassinosteroid; CESA, cellulose synthase; CSC, cellulose synthase complex; CWI, cell-wall integrity; OG, oligogalacturonide; PG, polygalacturonase; PME/PMEI, pectin methylesterase/inhibitor; RBOH, respiratory burst oxidase homolog; XEH, xyloglucan endohydrolase; XET, xyloglucan endotransglucosylase; XTH, xyloglucan endotransglucosylase/hydrolase.

**Table 2:** Phytohormonal control of cell-wall remodeling: mechanistic anchors and strength of evidence.

Hormone	Principal Wall-Level Outcome	Representative Mechanistic Basis	Representative Biological/Stress Context	References
Abscisic acid (ABA)	SCW thickening and lignification; endodermal suberization; guard-cell pectin remodeling.	SnRK2.2/2.3/2.6 phosphorylate NST1; ABA-responsive MYB41/53/92/93 promote suberin; PME53 modifies guard-cell pectin.	Drought/osmotic stress; salinity and root barrier control; stomatal/heat responses.	[13,42,74]
Auxin	Apoplastic acidification, wall loosening, and cell elongation.	TMK1/4 activate plasma-membrane H^+^-ATPases, lowering apoplastic pH and enabling expansin-mediated wall yielding.	Developmental/tropic elongation and environmentally responsive growth.	[3,11]
Ethylene	Context-dependent wall thickening, restricted elongation, or adaptive elongation.	Ethylene–EIN3 regulates *XTH33* under cadmium stress; submergence promotes apoplastic acidification and RpEXPA1 activity; in rice, OsEIL1 regulates OsCSLC-dependent wall establishment.	Cadmium-induced root growth inhibition; submergence escape growth; rice root growth regulation.	[14,87,88]
Brassinosteroids (BRs)	Cellulose biosynthesis, cell expansion, and pectin-dependent wall homeostasis.	BR-activated BES1 binds *CESA* promoters; pectin perturbation feeds back through BR signaling to restore wall homeostasis.	Growth coordination and compensatory CWI maintenance.	[12,77]
Jasmonates (JA)	SCW reinforcement, lignification, and CWI-linked growth/defense reprogramming.	Cellulose perturbation elevates JA; JA–MYC2 directly activates *MYB46* to promote SCW biosynthetic programs.	Wall damage, wounding, and stress-associated structural reinforcement.	[15,82]
Salicylic acid (SA)	CWI-associated defense signaling and phenylpropanoid/lignification responses.	Cellulose biosynthesis inhibition elevates SA and activates damage/immune transcriptional programs; CWI signaling converges with defense-associated lignification.	Cell-wall damage and immunity-associated reinforcement.	[15,16,81]
Gibberellins (GAs)	Rapid anisotropic cell expansion and elongation.	GA-triggered DELLA degradation releases growth programs that increase cell expansion; wall deposition/remodeling must remain coordinated with elongation.	Hypocotyl elongation; submergence-induced escape growth in rice.	[83,89]
Cytokinin (CK)	Growth cessation associated with increased wall stiffness.	CK promotes the elongation-to-differentiation transition; CK-dependent wall stiffening requires AUX1-mediated auxin input.	Root zonation and termination of cell elongation.	[84]
Strigolactones (SLs)	Cell elongation, cellulose deposition, and secondary-wall thickness.	In cotton fibers, SL signaling acts downstream of GAs to coordinate elongation and cellulose-dependent wall thickening.	Cotton fiber development; broader stress-linked wall functions remain less resolved.	[86]

## Data Availability

No new data were created or analyzed in this study. Data sharing is not applicable to this article.

## References

[1] Anderson C.T., Kieber J.J. (2020). Dynamic construction, perception, and remodeling of plant cell walls. Annu. Rev. Plant Biol..

[2] Vaahtera L., Schulz J., Hamann T. (2019). Cell wall integrity maintenance during plant development and interaction with the environment. Nat. Plants.

[3] Cosgrove D.J. (2024). Plant cell wall loosening by expansins. Annu. Rev. Cell Dev. Biol..

[4] Zhang B., Gao Y., Zhang L., Zhou Y. (2021). The plant cell wall: Biosynthesis, construction, and functions. J. Integr. Plant Biol..

[5] Tenhaken R. (2015). Cell wall remodeling under abiotic stress. Front. Plant Sci..

[6] Rui Y., Dinneny J.R. (2020). A wall with integrity: Surveillance and maintenance of the plant cell wall under stress. New Phytol..

[7] Gigli-Bisceglia N., Engelsdorf T., Hamann T. (2020). Plant cell wall integrity maintenance in model plants and crop species-relevant cell wall components and underlying guiding principles. Cell. Mol. Life Sci..

[8] Baez L.A., Tichá T., Hamann T. (2022). Cell wall integrity regulation across plant species. Plant Mol. Biol..

[9] Tang J., Guo H. (2025). Jack of all trades: Crosstalk between FERONIA signaling and hormone pathways. J. Exp. Bot..

[10] Xiong Y., Song X., Mehra P., Yu S., Li Q., Tashenmaimaiti D., Bennett M.J., Kong X., Bhosale R., Huang G. (2025). ABA-auxin cascade regulates crop root angle in response to drought. Curr. Biol..

[11] Lin W., Zhou X., Tang W., Takahashi K., Pan X., Dai J., Ren H., Zhu X., Pan S., Zheng H. (2021). TMK-based cell-surface auxin signalling activates cell-wall acidification. Nature.

[12] Wolf S., Mravec J., Greiner S., Mouille G., Höfte H. (2012). Plant cell wall homeostasis is mediated by brassinosteroid feedback signaling. Curr. Biol..

[13] Choi S.J., Lee Z., Kim S., Jeong E., Shim J.S. (2023). Modulation of lignin biosynthesis for drought tolerance in plants. Front. Plant Sci..

[14] Vreeburg R.A., Benschop J.J., Peeters A.J., Colmer T.D., Ammerlaan A.H., Staal M., Elzenga T.M., Staals R.H., Darley C.P., McQueen-Mason S.J. (2005). Ethylene regulates fast apoplastic acidification and expansin A transcription during submergence-induced petiole elongation in *Rumex palustris*. Plant J..

[15] Hamann T., Bennett M., Mansfield J., Somerville C. (2009). Identification of cell-wall stress as a hexose-dependent and osmosensitive regulator of plant responses. Plant J..

[16] Zhai K., Rhodes J., Zipfel C. (2024). A peptide-receptor module links cell wall integrity sensing to pattern-triggered immunity. Nat. Plants.

[17] Bacete L., Schulz J., Engelsdorf T., Bartosova Z., Vaahtera L., Yan G., Gerhold J.M., Tichá T., Øvstebø C., Gigli-Bisceglia N. (2022). THESEUS1 modulates cell wall stiffness and abscisic acid production in *Arabidopsis thaliana*. Proc. Natl. Acad. Sci. USA.

[18] McFarlane H.E. (2023). Open questions in plant cell wall synthesis. J. Exp. Bot..

[19] Scheller H.V., Ulvskov P. (2010). Hemicelluloses. Annu. Rev. Plant Biol..

[20] Sénéchal F., Wattier C., Rustérucci C., Pelloux J. (2014). Homogalacturonan-modifying enzymes: Structure, expression, and roles in plants. J. Exp. Bot..

[21] Anderson C.T., Pelloux J. (2025). The dynamics, degradation, and afterlives of pectins: Influences on cell wall assembly and structure, plant development and physiology, agronomy, and biotechnology. Annu. Rev. Plant Biol..

[22] Marjamaa K., Kukkola E.M., Fagerstedt K.V. (2009). The role of xylem class III peroxidases in lignification. J. Exp. Bot..

[23] Zhao Q., Nakashima J., Chen F., Yin Y., Fu C., Yun J., Shao H., Wang X., Wang Z.-Y., Dixon R.A. (2013). Laccase is necessary and nonredundant with peroxidase for lignin polymerization during vascular development in *Arabidopsis*. Plant Cell.

[24] Jalal A., Wang Y., Cai C., Ayaz A., Alabbosh K.F., Khan K.A., Han S., Zhu D. (2025). The architecture of adaptive lignin biosynthesis navigating environmental stresses in plants. J. Agron. Crop Sci..

[25] Peracchi L.M., Panahabadi R., Barros-Rios J., Bartley L.E., Sanguinet K.A. (2024). Grass lignin: Biosynthesis, biological roles, and industrial applications. Front. Plant Sci..

[26] Cosgrove D.J. (2022). Building an extensible cell wall. Plant Physiol..

[27] Oliveira D.M., Mota T.R., Salatta F.V., Sinzker R.C., Končitíková R., Kopečný D., Simister R., Silva M., Goeminne G., Morreel K. (2020). Cell wall remodeling under salt stress: Insights into changes in polysaccharides, feruloylation, lignification, and phenolic metabolism in maize. Plant Cell Environ..

[28] Tariq F., Ma C., Zhao S. (2025). Integrative dynamics of cell wall architecture and plant growth under salt stress. Front. Plant Sci..

[29] Hoffmann N., King S., Samuels A.L., McFarlane H.E. (2021). Subcellular coordination of plant cell wall synthesis. Dev. Cell.

[30] Gu Y., Rasmussen C.G. (2022). Cell biology of primary cell wall synthesis in plants. Plant Cell.

[31] Vellosillo T., Dinneny J.R., Somerville C.R., Ehrhardt D.W. (2021). TRANVIA (TVA) facilitates cellulose synthase trafficking and delivery to the plasma membrane. Proc. Natl. Acad. Sci. USA.

[32] Wang T., McFarlane H.E., Persson S. (2016). The impact of abiotic factors on cellulose synthesis. J. Exp. Bot..

[33] Zhong R., Ye Z.-H. (2015). Secondary cell walls: Biosynthesis, patterned deposition and transcriptional regulation. Crit. Rev. Plant Sci..

[34] Cosgrove D.J. (2024). Structure and growth of plant cell walls. Nat. Rev. Mol. Cell Biol..

[35] Rose J.K., Braam J., Fry S.C., Nishitani K. (2002). The XTH family of enzymes involved in xyloglucan endotransglucosylation and endohydrolysis: Current perspectives and a new unifying nomenclature. Crit. Rev. Plant Sci..

[36] Ishida K., Yokoyama R. (2022). Reconsidering the function of the xyloglucan endotransglucosylase/hydrolase family. J. Plant Res..

[37] Perrot T., Pauly M., Ramírez V. (2022). Emerging roles of β-glucanases in plant development and adaptative responses. Plants.

[38] Zhao N., Zhou Z., Cui S., Zhang X., Zhu S., Wang Y., Zenda T., Wenjing L. (2025). Advanced imaging-enabled understanding of cell wall remodeling mechanisms mediating plant drought stress tolerance. Front. Plant Sci..

[39] Yang J., Zhang Y., Jia J., Wang C., Fu Y. (2025). Flavonoid-lignin crosstalk: Engineering metabolic flux for optimised plant growth and stress resilience. Plant Cell Environ..

[40] Serra O., Geldner N. (2022). The making of suberin. New Phytol..

[41] Chen R., Wang P., Liu J., Yang X., Gong X., Zhou H., Han N., Yang Z. (2025). Suberin in plants: Biosynthesis, regulation, and its role in salt stress resistance. Front. Plant Sci..

[42] Shukla V., Han J.-P., Cléard F., Lefebvre-Legendre L., Gully K., Flis P., Berhin A., Andersen T.G., Salt D.E., Nawrath C. (2021). Suberin plasticity to developmental and exogenous cues is regulated by a set of MYB transcription factors. Proc. Natl. Acad. Sci. USA.

[43] Cantó-Pastor A., Kajala K., Shaar-Moshe L., Manzano C., Timilsena P., De Bellis D., Gray S., Holbein J., Yang H., Mohammad S. (2024). A suberized exodermis is required for tomato drought tolerance. Nat. Plants.

[44] McFarlane H.E., Döring A., Persson S. (2014). The cell biology of cellulose synthesis. Annu. Rev. Plant Biol..

[45] Li W., Wei J., Lei Y., Yang Z., Zhang S., Feng J., Li Y., Liu Y., Sheng H. (2025). Phosphorylation of cellulose synthases in plant responses to environmental changes. Int. J. Biol. Macromol..

[46] Chen J., Wan H., Zhao H., Dai X., Wu W., Liu J., Xu J., Yang R., Xu B., Zeng C. (2024). Identification and expression analysis of the Xyloglucan transglycosylase/hydrolase (XTH) gene family under abiotic stress in oilseed (*Brassica napus* L.). BMC Plant Biol..

[47] Bi H., Liu Z., Liu S., Qiao W., Zhang K., Zhao M., Wang D. (2024). Genome-wide analysis of wheat xyloglucan endotransglucosylase/hydrolase (XTH) gene family revealed TaXTH17 involved in abiotic stress responses. BMC Plant Biol..

[48] Pecatelli G., Caminada G., Coculo D., Lionetti V. (2026). Pectin under stress: Pectin methylesterases at the core of cell wall remodeling, integrity sensing, and signaling in plants. Plant Commun..

[49] Freitas C.D., Costa J.H., Germano T.A., Rocha R.d.O., Ramos M.V., Bezerra L.P. (2024). Class III plant peroxidases: From classification to physiological functions. Int. J. Biol. Macromol..

[50] Ao Y., Wu Q., Zheng J., Zhang C., Zhao Y., Xu R., Xue K., Dai C., Yang M. (2025). Building the physiological barrier: Suberin plasticity in response to environmental stimuli. Plant Sci..

[51] Yao X., Humphries J., Johnson K.L., Chen J., Ma Y. (2025). Function of WAKs in regulating cell wall development and responses to abiotic stress. Plants.

[52] Bawa G., Shen Y., Sun M., Sun X. (2025). Advances in CrRLK1L function in plant cell wall signaling during interaction with the environment and development. Stress Biol..

[53] Pu J., Ma J., Zhai H., Wu S., Wang Y., Putnis C.V., Wang L., Zhang W. (2025). Atomic force microscopy imaging of plant cell walls. Plant Physiol..

[54] Jarvis M.C. (2024). Forces on and in the cell walls of living plants. Plant Physiol..

[55] Monshausen G.B., Haswell E.S. (2013). A force of nature: Molecular mechanisms of mechanoperception in plants. J. Exp. Bot..

[56] Guichard M., Thomine S., Frachisse J.-M. (2022). Mechanotransduction in the spotlight of mechano-sensitive channels. Curr. Opin. Plant Biol..

[57] Franck C.M., Westermann J., Boisson-Dernier A. (2018). Plant malectin-like receptor kinases: From cell wall integrity to immunity and beyond. Annu. Rev. Plant Biol..

[58] Cheung A.Y. (2024). FERONIA: A receptor kinase at the core of a global signaling network. Annu. Rev. Plant Biol..

[59] Feng W., Kita D., Peaucelle A., Cartwright H.N., Doan V., Duan Q., Liu M.-C., Maman J., Steinhorst L., Schmitz-Thom I. (2018). The FERONIA receptor kinase maintains cell-wall integrity during salt stress through Ca^2+^ signaling. Curr. Biol..

[60] Lin W., Tang W., Pan X., Huang A., Gao X., Anderson C.T., Yang Z. (2022). *Arabidopsis* pavement cell morphogenesis requires FERONIA binding to pectin for activation of ROP GTPase signaling. Curr. Biol..

[61] Hématy K., Sado P.-E., Van Tuinen A., Rochange S., Desnos T., Balzergue S., Pelletier S., Renou J.-P., Höfte H. (2007). A receptor-like kinase mediates the response of *Arabidopsis* cells to the inhibition of cellulose synthesis. Curr. Biol..

[62] Gonneau M., Desprez T., Martin M., Doblas V.G., Bacete L., Miart F., Sormani R., Hématy K., Renou J., Landrein B. (2018). Receptor kinase THESEUS1 is a rapid alkalinization factor 34 receptor in *Arabidopsis*. Curr. Biol..

[63] Van der Does D., Boutrot F., Engelsdorf T., Rhodes J., McKenna J.F., Vernhettes S., Koevoets I., Tintor N., Veerabagu M., Miedes E. (2017). The *Arabidopsis* leucine-rich repeat receptor kinase MIK2/LRR-KISS connects cell wall integrity sensing, root growth and response to abiotic and biotic stresses. PLoS Genet..

[64] Kohorn B.D., Johansen S., Shishido A., Todorova T., Martinez R., Defeo E., Obregon P. (2009). Pectin activation of MAP kinase and gene expression is WAK2 dependent. Plant J..

[65] Kohorn B.D. (2016). Cell wall-associated kinases and pectin perception. J. Exp. Bot..

[66] Haruta M., Sabat G., Stecker K., Minkoff B.B., Sussman M.R. (2014). A peptide hormone and its receptor protein kinase regulate plant cell expansion. Science.

[67] Xiao Y., Stegmann M., Han Z., DeFalco T.A., Parys K., Xu L., Belkhadir Y., Zipfel C., Chai J. (2019). Mechanisms of RALF peptide perception by a heterotypic receptor complex. Nature.

[68] Zhao C., Zayed O., Yu Z., Jiang W., Zhu P., Hsu C.-C., Zhang L., Tao W.A., Lozano-Durán R., Zhu J.-K. (2018). Leucine-rich repeat extensin proteins regulate plant salt tolerance in *Arabidopsis*. Proc. Natl. Acad. Sci. USA.

[69] Zhao C., Jiang W., Zayed O., Liu X., Tang K., Nie W., Li Y., Xie S., Li Y., Long T. (2021). The LRXs-RALFs-FER module controls plant growth and salt stress responses by modulating multiple plant hormones. Natl. Sci. Rev..

[70] Debnath J., Morton R.N., Engelsdorf T., Gigli-Bisceglia N. (2026). Plant cell wall remodeling and peptide signaling under abiotic and biotic stress. Plant Commun..

[71] Duan Q., Kita D., Li C., Cheung A.Y., Wu H.-M. (2010). FERONIA receptor-like kinase regulates RHO GTPase signaling of root hair development. Proc. Natl. Acad. Sci. USA.

[72] Fichman Y., Mittler R. (2020). Rapid systemic signaling during abiotic and biotic stresses: Is the ROS wave master of all trades?. Plant J..

[73] Huang Y.-C., Wu H.-C., Wang Y.-D., Liu C.-H., Lin C.-C., Luo D.-L., Jinn T.-L. (2017). PECTIN METHYLESTERASE34 contributes to heat tolerance through its role in promoting stomatal movement. Plant Physiol..

[74] Wu H.-C., Yu S.-Y., Wang Y.-D., Jinn T.-L. (2022). Guard cell-specific pectin METHYLESTERASE53 is required for abscisic acid-mediated stomatal function and heat response in *Arabidopsis*. Front. Plant Sci..

[75] Jobert F., Yadav S., Robert S. (2023). Auxin as an architect of the pectin matrix. J. Exp. Bot..

[76] Hattori Y., Nagai K., Furukawa S., Song X.-J., Kawano R., Sakakibara H., Wu J., Matsumoto T., Yoshimura A., Kitano H. (2009). The ethylene response factors SNORKEL1 and SNORKEL2 allow rice to adapt to deep water. Nature.

[77] Xie L., Yang C., Wang X. (2011). Brassinosteroids can regulate cellulose biosynthesis by controlling the expression of CESA genes in *Arabidopsis*. J. Exp. Bot..

[78] Percio F., Rubio L., Amorim-Silva V., Botella M.A. (2025). Crucial roles of brassinosteroids in cell wall composition and structure across species: New insights and biotechnological applications. Plant Cell Environ..

[79] Caño-Delgado A., Penfield S., Smith C., Catley M., Bevan M. (2003). Reduced cellulose synthesis invokes lignification and defense responses in *Arabidopsis thaliana*. Plant J..

[80] Munzert K.S., Engelsdorf T. (2025). Plant cell wall structure and dynamics in plant–pathogen interactions and pathogen defence. J. Exp. Bot..

[81] Wang M., Li Z., Ran M., Han Y., Liu X., Zhao C. (2026). Plant cell wall signaling: From perception to adaptive responses. J. Genet. Genom..

[82] Im J.H., Son S., Kim W.C., Kim K., Mitsuda N., Ko J.H., Han K.H. (2024). Jasmonate activates secondary cell wall biosynthesis through MYC2-MYB46 module. Plant J..

[83] Sauret-Güeto S., Calder G., Harberd N.P. (2012). Transient gibberellin application promotes *Arabidopsis thaliana* hypocotyl cell elongation without maintaining transverse orientation of microtubules on the outer tangential wall of epidermal cells. Plant J..

[84] Liu S., Strauss S., Adibi M., Mosca G., Yoshida S., Ioio R.D., Runions A., Andersen T.G., Grossmann G., Huijser P. (2022). Cytokinin promotes growth cessation in the *Arabidopsis* root. Curr. Biol..

[85] Samalova M., Melnikava A., Elsayad K., Peaucelle A., Gahurova E., Gumulec J., Spyroglou I., Zemlyanskaya E.V., Ubogoeva E.V., Balkova D. (2024). Hormone-regulated expansins: Expression, localization, and cell wall biomechanics in *Arabidopsis* root growth. Plant Physiol..

[86] Wen Y., He P., Bai X., Zhang H., Zhang Y., Yu J. (2024). Strigolactones modulate cotton fiber elongation and secondary cell wall thickening. J. Integr. Agric..

[87] Zhou Y., Gao Y.-H., Zhang B.-C., Yang H.-L., Tian Y.-B., Huang Y.-H., Yin C.-C., Tao J.-J., Wei W., Zhang W.-K. (2024). CELLULOSE SYNTHASE-LIKE C proteins modulate cell wall establishment during ethylene-mediated root growth inhibition in rice. Plant Cell.

[88] Kong X., Li C., Zhang F., Yu Q., Gao S., Zhang M., Tian H., Zhang J., Yuan X., Ding Z. (2018). Ethylene promotes cadmium-induced root growth inhibition through EIN3 controlled XTH33 and LSU1 expression in *Arabidopsis*. Plant Cell Environ..

[89] Ashikari M., Nagai K., Bailey-Serres J. (2025). Surviving floods: Escape and quiescence strategies of rice coping with submergence. Plant Physiol..

[90] Kim G., Ryu H., Sung J. (2022). Hormonal crosstalk and root suberization for drought stress tolerance in plants. Biology.

[91] Chen Y., Zhang H., Chen W., Gao Y., Xu K., Sun X., Huo L. (2024). The role of ethylene in the regulation of plant response mechanisms to waterlogging stress. Plant Cell Rep..

[92] Yeung E., Bailey-Serres J., Sasidharan R. (2019). After the deluge: Plant revival post-flooding. Trends Plant Sci..

[93] Cheng Z., Wang C., Tang F., Zhou Y., Zhu C., Ding Y. (2025). The cell wall functions in plant heavy metal response. Ecotoxicol. Environ. Saf..

[94] Neres D.F., Wright R.C. (2024). Pleiotropy, a feature or a bug? Toward co-ordinating plant growth, development, and environmental responses through engineering plant hormone signaling. Curr. Opin. Biotechnol..

[95] Bhar A., Jokipii-Lukkari S., Teeri T.H., Kärkönen A. (2026). Hormonal crosstalk in the regulation of lignification: Where do we stand?. J. Exp. Bot..

[96] Gall H.L., Philippe F., Domon J.-M., Gillet F., Pelloux J., Rayon C. (2015). Cell wall metabolism in response to abiotic stress. Plants.

[97] Jiang Z., van Zanten M., Sasidharan R. (2025). Mechanisms of plant acclimation to multiple abiotic stresses. Commun. Biol..

[98] Aswathi K., Ul-Allah S., Puthur J.T., Siddique K.H., Frei M., Farooq M. (2025). The plant mind: Unraveling abiotic stress priming, memory, and adaptation. Physiol. Plant..

[99] Ma L., Xing L., Li Z., Jiang D. (2025). Epigenetic control of plant abiotic stress responses. J. Genet. Genom..

[100] Jeong H.J., Jung K.H. (2015). Rice tissue-specific promoters and condition-dependent promoters for effective translational application. J. Integr. Plant Biol..

[101] Shi L., Su J., Cho M.-J., Song H., Dong X., Liang Y., Zhang Z. (2023). Promoter editing for the genetic improvement of crops. J. Exp. Bot..

[102] Lozano-Juste J., Infantes L., Garcia-Maquilon I., Ruiz-Partida R., Merilo E., Benavente J.L., Velazquez-Campoy A., Coego A., Bono M., Forment J. (2023). Structure-guided engineering of a receptor-agonist pair for inducible activation of the ABA adaptive response to drought. Sci. Adv..

[103] Barbut F.R., Cavel E., Donev E.N., Gaboreanu I., Urbancsok J., Pandey G., Demailly H., Jiao D., Yassin Z., Derba-Maceluch M. (2024). Integrity of xylan backbone affects plant responses to drought. Front. Plant Sci..

[104] Lu C., Li W., Feng X., Chen J., Hu S., Tan Y., Wu L. (2025). The dynamic remodeling of plant cell wall in response to heat stress. Genes.

[105] Wu H.-C., Hsu S.-F., Luo D.-L., Chen S.-J., Huang W.-D., Lur H.-S., Jinn T.-L. (2010). Recovery of heat shock-triggered released apoplastic Ca^2+^ accompanied by pectin methylesterase activity is required for thermotolerance in soybean seedlings. J. Exp. Bot..

[106] Amsbury S., Hunt L., Elhaddad N., Baillie A., Lundgren M., Verhertbruggen Y., Scheller H.V., Knox J.P., Fleming A.J., Gray J.E. (2016). Stomatal function requires pectin de-methyl-esterification of the guard cell wall. Curr. Biol..

[107] Sharma L., Priya M., Kaushal N., Bhandhari K., Chaudhary S., Dhankher O.P., Prasad P.V., Siddique K.H., Nayyar H. (2020). Plant growth-regulating molecules as thermoprotectants: Functional relevance and prospects for improving heat tolerance in food crops. J. Exp. Bot..

[108] Liu M.-C.J., Yeh F.-L.J., Yvon R., Simpson K., Jordan S., Chambers J., Wu H.-M., Cheung A.Y. (2024). Extracellular pectin-RALF phase separation mediates FERONIA global signaling function. Cell.

[109] Ullah S., Matin M.N., Yin C., Mas-Ud M.A., Khan A., Islam M.S., Irfanullah, Haq I.u. (2026). Phytohormone-Mediated Regulation of Plant Cold Stress Tolerance: Signaling, Hormonal Crosstalk, and Translational Perspectives. Int. J. Mol. Sci..

[110] Sha S., Zeng C., Shang X., Zou B., Sun T., Yang Y. (2026). Root morphogenetic responses to waterlogging stress in plants: From structural reconfiguration to molecular regulatory mechanisms. Front. Plant Sci..

[111] Nagai K., Ashikari M. (2023). Molecular mechanism of internode elongation in rice. Breed. Sci..

[112] Li J., Fan H., Song Q., Jing L., Yu H., Li R., Zhang P., Liu F., Li W., Sun L. (2023). Physiological and molecular bases of the boron deficiency response in tomatoes. Hortic. Res..

[113] Clúa J., Montpetit J., Jimenez-Sandoval P., Naumann C., Santiago J., Poirier Y. (2024). A CYBDOM protein impacts iron homeostasis and primary root growth under phosphate deficiency in *Arabidopsis*. Nat. Commun..

[114] Leal A.R., Belo J., Beeckman T., Barros P.M., Oliveira M.M. (2022). The combined effect of heat and osmotic stress on suberization of *Arabidopsis* roots. Cells.

[115] Hu W., Gao M., Xu B., Wang S., Wang Y., Zhou Z. (2022). Co-occurring elevated temperature and drought stresses during cotton fiber thickening stage inhibit fiber biomass accumulation and cellulose synthesis. Ind. Crops Prod..

[116] Kumari S., Basu S., Kumar G. (2024). A systematic review on the implications of concurrent heat and drought stress in modulating floral development in plants. Plant Sci..

[117] Li N., Geng Z., Huang X., Huang S., Song L., Chen R., Chen Z., Du L., Xu C. (2025). A review of differential plant responses to drought, heat, and combined drought+ heat stress. Curr. Issues Mol. Biol..

[118] Roig-Oliver M., Bresta P., Nikolopoulos D., Bota J., Flexas J. (2025). Dynamics of pectins and photosynthetic traits in sunflower and tomato subjected to gradual recoveries preceded by water deprivation. Physiol. Plant..

[119] Chun H.J., Baek D., Cho H.M., Jung H.S., Jeong M.S., Jung W.-H., Choi C.W., Lee S.H., Jin B.J., Park M.S. (2019). Metabolic adjustment of *Arabidopsis* root suspension cells during adaptation to salt stress and mitotic stress memory. Plant Cell Physiol..

[120] Chen Y., Xia P. (2025). NAC transcription factors as biological macromolecules responded to abiotic stress: A comprehensive review. Int. J. Biol. Macromol..

[121] Amin N., Lu L., Rehman F., Imran M., Gai Y., Wang P., Jian W. (2025). Transcriptional regulation of GmNAC3-mediated drought stress tolerance in soybean. GM Crops Food.

[122] Tuncel A., Pan C., Clem J.S., Liu D., Qi Y. (2025). CRISPR–Cas applications in agriculture and plant research. Nat. Rev. Mol. Cell Biol..

[123] Zaid A., Singh A., Uddin K. (2026). Epigenetic mechanism of abiotic stress responses in plants. Horticulturae.

[124] Yu G., Xiang J., Lai C., Li X., Sunahara G.I., Mo F., Zhang X., Liu J., Lin H., Liu G. (2025). Unveiling the spatiotemporal strategies of plants in response to biotic and abiotic stresses: A comprehensive review. Plant Physiol. Biochem..

[125] Usman B., Khan N., Wang Y., Gupta R., Kwon S.W., Kim S.T. (2025). Probing cellular landscapes in plants via single-cell transcriptomics. Plant Sci..

[126] Barmukh R., Garg V., Liu H., Chitikineni A., Xin L., Henry R., Varshney R.K. (2025). Spatial omics for accelerating plant research and crop improvement. Trends Biotechnol..

[127] Xu P., Wang B., Hu Y., La Q., Zhang T. (2026). Advances in single-cell and spatial multi-omics for plant development and stress responses. Plant Divers..

[128] Lou H., Li S., Shi Z., Zou Y., Zhang Y., Huang X., Yang D., Yang Y., Li Z., Xu C. (2025). Engineering source-sink relations by prime editing confers heat-stress resilience in tomato and rice. Cell.

[129] Schreiber M., Jayakodi M., Stein N., Mascher M. (2024). Plant pangenomes for crop improvement, biodiversity and evolution. Nat. Rev. Genet..

[130] Li T., Mohamedikbal S., Bestry M., Batley J., Edwards D. (2025). Pangenomics combined with artificial intelligence and precision breeding can accelerate crop improvement. Curr. Opin. Plant Biol..

[131] Szabados L., Kant K., Rabilu S., Rashid A., Zsigmond L. (2026). Crops exposed to extreme conditions: Perspectives of gene editing to improve stress tolerance. Plant Cell Rep..

[132] Prado K., Holland B.L., McSpadden Gardener B., Lundquist P.K., Santiago J.P., VanBuren R., Rhee S.Y. (2025). Building climate-resilient crops: Genetic, environmental, and technological strategies for heat and drought stress tolerance. J. Exp. Bot..

[133] Saini D.K., Lal M.K., Singh M.P., Jagadish S.K. (2025). Translational research for climate-ready agriculture. Plant Physiol. Rep..

[134] Ha C.M., Rao X., Saxena G., Dixon R.A. (2021). Growth–defense trade-offs and yield loss in plants with engineered cell walls. New Phytol..

